# The rs7799039 variant in the leptin gene promoter drives insulin resistance through reduced serum leptin levels

**DOI:** 10.3389/fendo.2025.1589575

**Published:** 2025-10-13

**Authors:** Yongyan Song, Youjin Zhang, Xinyu Liu, Wang Gong, Yinquan Ai, Binger Shen, Jing Li, Chuan He

**Affiliations:** ^1^ Central Laboratory, Clinical Medical College & Affiliated Hospital of Chengdu University, Chengdu, Sichuan, China; ^2^ Clinical Medical College of Chengdu University, Chengdu, Sichuan, China; ^3^ Department of Cardiology, Clinical Medical College & Affiliated Hospital of Chengdu University, Chengdu, Sichuan, China

**Keywords:** leptin, rs7799039, rs1137101, rs1805094, glucose-lipid metabolism, variant

## Abstract

**Background:**

The associations between the rs7799039 variant in the promoter region of the leptin gene (*LEP*) and the rs1137100, rs1137101, and rs1805094 variants in the exons of the leptin receptor gene (*LEPR*) with leptin levels and glucose-lipid metabolism markers have been examined across various populations. However, the findings have been inconsistent and, at times, contradictory.

**Methods:**

Eligible studies were identified through a search of PubMed, Google Scholar, Embase, Cochrane Library, Web of Science, CNKI, Wanfang, and VIP databases. A random-effects model was employed, and the standardized mean difference (SMD) with 95% confidence interval (95% CI) was calculated to assess the differences in leptin levels and glucose-lipid metabolism markers between subjects with different genotypes of the rs7799039, rs1137100, rs1137101, or rs1805094 variants. Heterogeneity among studies was evaluated using Cochran’s Q-test, based on the χ² statistic. Publication bias was assessed using Begg’s test.

**Results:**

A total of 33 studies (10,471 subjects) for the rs7799039 variant, 12 studies (6,595 subjects) for the rs1137100 variant, 48 studies (18,890 subjects) for the rs1137101 variant, and 20 studies (5,051 subjects) for the rs1805094 variant were included in the pooled analyses. A significant association was found for A-allele carriers of *LEP* rs7799039 variant, who exhibited lower levels of leptin (SMD = -0.18 ng/mL, 95% CI = -0.31 to -0.04 ng/mL, *p* = 0.01), and higher levels of insulin (SMD = 0.22 μmol/μL, 95% CI = 0.07 to 0.37 μmol/μL, *p* < 0.01), and HOMA-IR (SMD = 0.26, 95% CI = 0.08 to 0.43, *p* < 0.01) compared to GG homozygotes. For *LEPR* rs1137100, rs1137101, and rs1805094 variants, no significant associations with leptin or glucose-lipid metabolism markers were observed in the pooled meta-analyses of the total population. However, significant associations were detected between the rs1137101 and rs1805094 variants and leptin or glucose-lipid metabolism markers in subgroup analyses stratified by sex, ethnicity, and health status.

**Conclusions:**

The meta-analysis suggests that the A allele of *LEP* rs7799039 variant is associated with an increased risk of insulin resistance, potentially through its effect on reducing leptin levels. *LEPR* rs1137101 and rs1805094 variants show weak associations with leptin levels and glucose-lipid metabolism markers.

**Systematic review registration:**

, identifier CRD42025373543.

## Introduction

Glycolipid metabolic disorders, including abnormalities in lipid and glucose metabolism, represent a significant risk factor for the development of diabetes and cardiovascular disease (CVD) (1, 2). These disorders are responsible for at least 50% of the population-attributable risk for CVD, making them a key focus in cardiovascular health research (2). Gene variants, which refer to variations in DNA sequences, play a crucial role in the onset, progression, and severity of glycolipid metabolic disorders (3–10). These genetic variations can affect the expression of the genes implicated in lipid and glucose metabolism, influencing individual susceptibility to conditions such as hyperglycemia, hyperlipidemia, and metabolic syndrome (5, 9). Among the most widely studied variants in relation to glycolipid metabolic disorders are those located in the genes of adipokines and their receptors.

Leptin is an important member of adipokines. It is often referred to as the “satiety hormone” because it helps signal the brain to reduce appetite and increase energy expenditure when fat stores are sufficient (11). The gene encoding leptin is called *LEP* and is located on chromosome 7 in humans. The rs7799039 variant, situated in the 5’ promoter region of the *LEP* gene, is formed by a single-nucleotide variation from guanine (G) to adenine (A). The A allele of rs7799039 has been associated with an increased risk of glycolipid metabolic disorders (12, 13). Leptin exerts its physiological function by combining with the leptin receptor (LEPR), which is primarily located in the hypothalamus of the brain but is also found in other tissues throughout the body. The *LEPR* gene is located on chromosome 1 at position 1p31. Three missense mutations in the *LEPR* gene—rs1137100 (Lys109Arg), rs1137101 (Gln223Arg), and rs1805094 (Lys656Asn)—have been linked to a higher risk of glycolipid metabolic disorders (14–16).

However, there are numerous inconsistencies and contradictions in the published data regarding these four polymorphic loci and glucose-lipid metabolism markers. Some studies have shown that the A allele of the rs7799039 variant is associated with significantly higher levels of glucose (17), insulin (18), homeostasis model assessment of insulin resistance (HOMA-IR) (18), triglycerides (19), total cholesterol (TC) (20), low-density lipoprotein cholesterol (LDL-C) (21), and a lower level of high-density lipoprotein cholesterol (HDL-C) (22), whereas other studies have not supported these findings (23, 24). Additionally, there is significant inconsistency in the published data concerning the relationships between the rs1137100, rs1137101, and rs1805094 variants and glucose-lipid metabolism markers. Some studies have indicated that the A allele of rs1137100, G allele of rs1137101, and C allele of rs1805094 are linked to significantly higher levels of glucose (25–27), insulin (25, 26, 28), HOMA-IR (14, 26, 29), triglycerides (30–32), TC (26, 29, 30), LDL-C (29, 30, 33), and lower levels of HDL-C (26, 28, 34), while other studies have found no significant associations between these variants and glucose-lipid metabolism markers (35–37).

In this study, a systematic review and meta-analysis was conducted using previous publications to explore the relationships between the rs7799039, rs1137100, rs1137101, or rs1805094 variants and leptin and glucose-lipid metabolism markers. The results offer an opportunity to unveil the interrelationships among these variants, leptin, glycolipid metabolic disorders, and CVD.

## Methods

### Literature search strategy

This meta-analysis was registered in PROSPERO (CRD42025373543) and was carried out in accordance with the Preferred Reporting Items for Systematic Reviews and Meta-Analyses (PRISMA) guidelines. A thorough search was conducted across multiple databases, including PubMed, Google Scholar, Embase, Cochrane Library, Web of Science, CNKI, Wanfang, and VIP, from their inception up until February 2025. The search utilized the following keywords: (“leptin” or “LEP”) and (“leptin receptor” or “LEPR”) and (“glucose” or “insulin” or “homeostasis model assessment of insulin resistance” or “HOMA-IR”) and (“triglycerides” or “total cholesterol” or “low-density lipoprotein cholesterol” or “high-density lipoprotein cholesterol” or “TC” or “LDL-C” or “HDL-C”). The variables considered in this meta-analysis include leptin, glucose metabolism markers (glucose, insulin, and HOMA-IR) and lipid metabolism markers (triglycerides, TC, LDL-C, and HDL-C). All studies that reported on the associations between the rs7799039, rs1137100, rs1137101, or rs1805094 variants and any of the eight indexes were reviewed and assessed.

### Inclusion and exclusion criteria

Inclusion criteria: 1) The sample size and genotype distribution were clearly and accurately reported; 2) At least one of the eight variables (leptin, glucose, insulin, HOMA-IR, triglycerides, TC, LDL-C, and HDL-C) was provided; 3) Data were presented as mean ± standard deviation or mean ± standard error. Exclusion criteria: 1) Articles published more than once; 2) Incomplete data; 3) Case reports; 4) Conference abstracts; 5) Animal studies.

### Data extraction

Data were independently extracted by two reviewers. The information collected from each study included the first author’s name, year of publication, ethnicity, age, gender, health status, sample size, and mean ± standard deviation/standard error by genotype. If standard error was provided, standard deviation was calculated. The units for the indexes were ng/mL (leptin), μmol/μL (insulin), and mg/dL (glucose, triglycerides, TC, LDL-C, and HDL-C), with unit conversions made where other units were used. The data were thoroughly checked for accuracy, and any discrepancies were resolved through group discussion.

### Meta-analysis

The meta-analysis was conducted using the STATA software package version 10.1 (STATA, College Station, TX, USA). A dominant model was applied since most of the studies included in the analysis presented results in a dominant fashion (i.e., [AA + AG] vs. GG for rs7739039, [GG + AG] vs. AA for rs1137100, [GG + AG] vs. AA for rs1137101, and [CC + CG] vs. GG for rs1805094). If a study contained multiple subgroups (e.g., based on gender, age, ethnicity, or health condition), each subgroup was treated as an independent comparison in the meta-analysis. Subgroup analyses were performed with at least three comparisons to ensure sufficient statistical power. The standardized mean difference (SMD) and 95% confidence interval (95% CI) were used to evaluate differences in leptin and glucose-lipid metabolism markers between the genotypes of the rs7799039, rs1137100, rs1137101, or rs1805094 variants. A random-effects model was chosen for the meta-analysis as it tends to provide more conservative results than the fixed-effects model. Heterogeneity among studies was assessed using Cochran’s x^2^-based Q-statistic test, and Galbraith plots were employed to identify potential sources of heterogeneity. Subgroup analyses were carried out, with subgroups categorized by gender (males and females), age (adults and children/adolescents), ethnicity (Caucasians, Latin Americans, Asians, and Africans), and health condition (overweight/obesity, type 2 diabetes mellitus [T2DM], hypertension, and general/control subjects). Publication bias was evaluated using Begg’s test and visualized through Begg’s funnel plot. If publication bias was detected, the trim-and-fill method was applied to adjust the results. All *p*-values were two-tailed, with *p* ≤ 0.05 considered statistically significant.

## Results

### Characteristics of the enrolled studies

**Figure 1:**
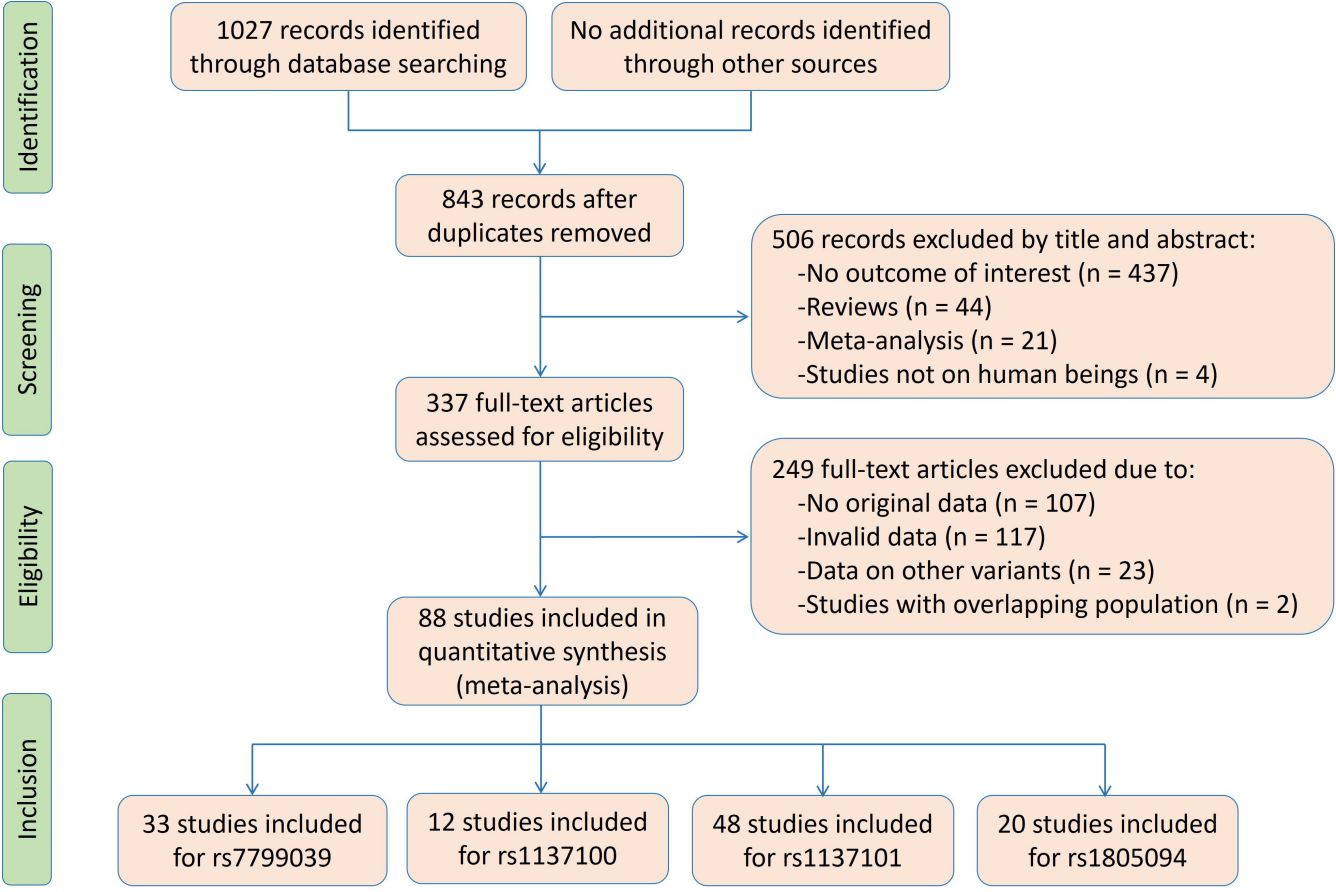
The flow diagram of the literature search.

The flow diagram of the literature search is shown in **Figure 1**. Thirty-three studies, 12 studies, 48 studies and 20 studies were respectively enrolled for the rs7799039, rs1137100, rs1137101 and rs1805094 variants, and the reference list of the included studies is presented in **Supplementary Table S1**.

Characteristics of the enrolled studies for *LEP* rs7799039 variant are shown in **Supplementary Table S2**. The enrolled articles were published between 2007 and 2022, and written either in English (28 articles) or in Chinese (5 articles). Five studies, 4 studies, 18 studies and 6 studies involved Caucasians, Latin Americans, Asians and Africans, respectively. Eleven studies and 6 studies involved overweight/obesity, and T2DM, respectively. Four study only involved males, 2 studies only involved females, and the rest studies involved both genders. The subjects from 9 studies were divided into subgroups according to gender, age, ethnicity, and health condition, and each subgroup was treated as an independent comparison. Original data for leptin and glucose-lipid metabolism markers by genotypes of the rs7799039 variant are shown in **Supplementary Tables S3** and **S4**. Seventeen studies, 17 studies, 13 studies, 13 studies, 19 studies, 19 studies, 19 studies and 20 studies presented the data for leptin, glucose, insulin, HOMA-IR, triglycerides, TC, LDL-C and HDL-C, respectively. A total of 43 comparisons were distinguished among the 33 studies included for the rs7799039 variant, and 21 comparisons, 24 comparisons, 20 comparisons, 18 comparisons, 26 comparisons, 26 comparisons, 26 comparisons and 27 comparisons were enrolled to compare the differences in leptin, glucose, insulin, HOMA-IR, triglycerides, TC, LDL-C and HDL-C, respectively. Ten thousand four hundred and seventy-one subjects were included in the meta-analysis for the rs7799039 variant. Among these subjects, 2,914 subjects (27.83%) had the GG genotype, and 7,557 subjects (72.17%) had the AA or AG genotype.

Characteristics of the enrolled studies for *LEPR* rs1137100 variant are shown in **Supplementary Table S5**. The enrolled articles were published between 2000 and 2016, and written either in English (9 articles) or in Chinese (3 articles). Eight studies and 4 studies involved Caucasians and Asians, respectively. Four studies, and 2 studies involved overweight/obesity, impaired glucose tolerance, respectively. One study only involved males, 2 studies only involved females, and the rest studies involved both genders. The subjects from 2 studies were divided into subgroups according to gender, age, ethnicity, and health condition, and each subgroup was treated as an independent comparison. Original data for leptin and glucose-lipid metabolism markers by genotypes of the rs1137100 variant are shown in **Supplementary Tables S6** and **S7**. Seven studies, 7 studies, 6 studies, 4 studies, 6 studies, 7 studies, 7 studies and 6 studies presented the data for leptin, glucose, insulin, HOMA-IR, triglycerides, TC, LDL-C and HDL-C, respectively. A total of 14 comparisons were distinguished among the 12 studies included for the rs1137100 variant, and 8 comparisons, 8 comparisons, 7 comparisons, 4 comparisons, 6 comparisons, 7 comparisons, 7 comparisons and 6 comparisons were enrolled to compare the differences in leptin, glucose, insulin, HOMA-IR, triglycerides, TC, LDL-C and HDL-C, respectively. Six thousand five hundred and ninety-five subjects were included in the meta-analysis for the rs1137100 variant. Among these subjects, 1,956 subjects (29.66%) had the AA genotype, and 4,639 subjects (70.34%) had the GG or AG genotype.

Characteristics of the enrolled studies for *LEPR* rs1137101 variant are shown in **Supplementary Table S8**. The enrolled articles were published between 1997 and 2022, and written either in English (40 articles) or in Chinese (8 articles). Nineteen studies, 7 studies, 18 studies and 4 studies involved Caucasians, Latin Americans, Asians and Africans, respectively. Nineteen studies, 4 studies, and 4 studies involved overweight/obesity, T2DM and hypertension, respectively. Ten studies only involved males, 12 studies only involved females, and the rest studies involved both genders. The subjects from 18 studies were divided into subgroups according to gender, age, ethnicity, and health condition, and each subgroup was treated as an independent comparison. Original data for leptin and glucose-lipid metabolism markers by genotypes of the rs1137101 variant are shown in **Supplementary Tables S9** and **S10**. Thirty studies, 26 studies, 18 studies, 13 studies, 25 studies, 27 studies, 24 studies and 27 studies presented the data for leptin, glucose, insulin, HOMA-IR, triglycerides, TC, LDL-C and HDL-C, respectively. A total of 69 comparisons were distinguished among the 48 studies included for the rs1137101 variant, and 43 comparisons, 33 comparisons, 23 comparisons, 17 comparisons, 33 comparisons, 35 comparisons, 33 comparisons and 36 comparisons were enrolled to compare the differences in leptin, glucose, insulin, HOMA-IR, triglycerides, TC, LDL-C and HDL-C, respectively. Eighteen thousand eight hundred and ninety subjects were included in the meta-analysis for the rs1137101 variant. Among these subjects, 5,587 subjects (29.58%) had the AA genotype, and 13,303 subjects (70.42%) had the GG or AG genotype.

Characteristics of the enrolled studies for the rs1805094 variant are shown in **Supplementary Table S11**. The enrolled articles were published between 2000 and 2020, and all of them were written in English. Fifteen studies, 2 studies and 3 studies involved Caucasians, Latin Americans and Asians, respectively. Eleven studies and 3 studies involved overweight/obesity and T2DM, respectively. Six studies only involved males, 6 studies only involved females, and the rest studies involved both genders. The subjects from 9 studies were divided into subgroups according to gender, age, ethnicity, and health condition, and each subgroup was treated as an independent comparison. Original data for leptin and glucose-lipid metabolism markers by genotypes of the rs1805094 variant are shown in **Supplementary Tables S12** and **S13**. Fifteen studies, 11 studies, 11 studies, 7 studies, 12 studies, 11 studies, 11 studies and 11 studies presented the data for leptin, glucose, insulin, HOMA-IR, triglycerides, TC, LDL-C and HDL-C, respectively. A total of 29 comparisons were distinguished among the 20 studies included for the rs1137101 variant, and 21 comparisons, 15 comparisons, 16 comparisons, 9 comparisons, 16 comparisons, 14 comparisons, 15 comparisons and 15 comparisons were enrolled to compare the differences in leptin, glucose, insulin, HOMA-IR, triglycerides, TC, LDL-C and HDL-C, respectively. Five thousand fifty-one subjects were included in the meta-analysis for the rs1137101 variant. Among these subjects, 3,430 subjects (67.91%) had the GG genotype, and 1,621 subjects (32.09%) had the CC or CG genotype.

### Associations of *LEP* rs7739039 variant with leptin and glucose-lipid metabolism markers

**Figure 2:**
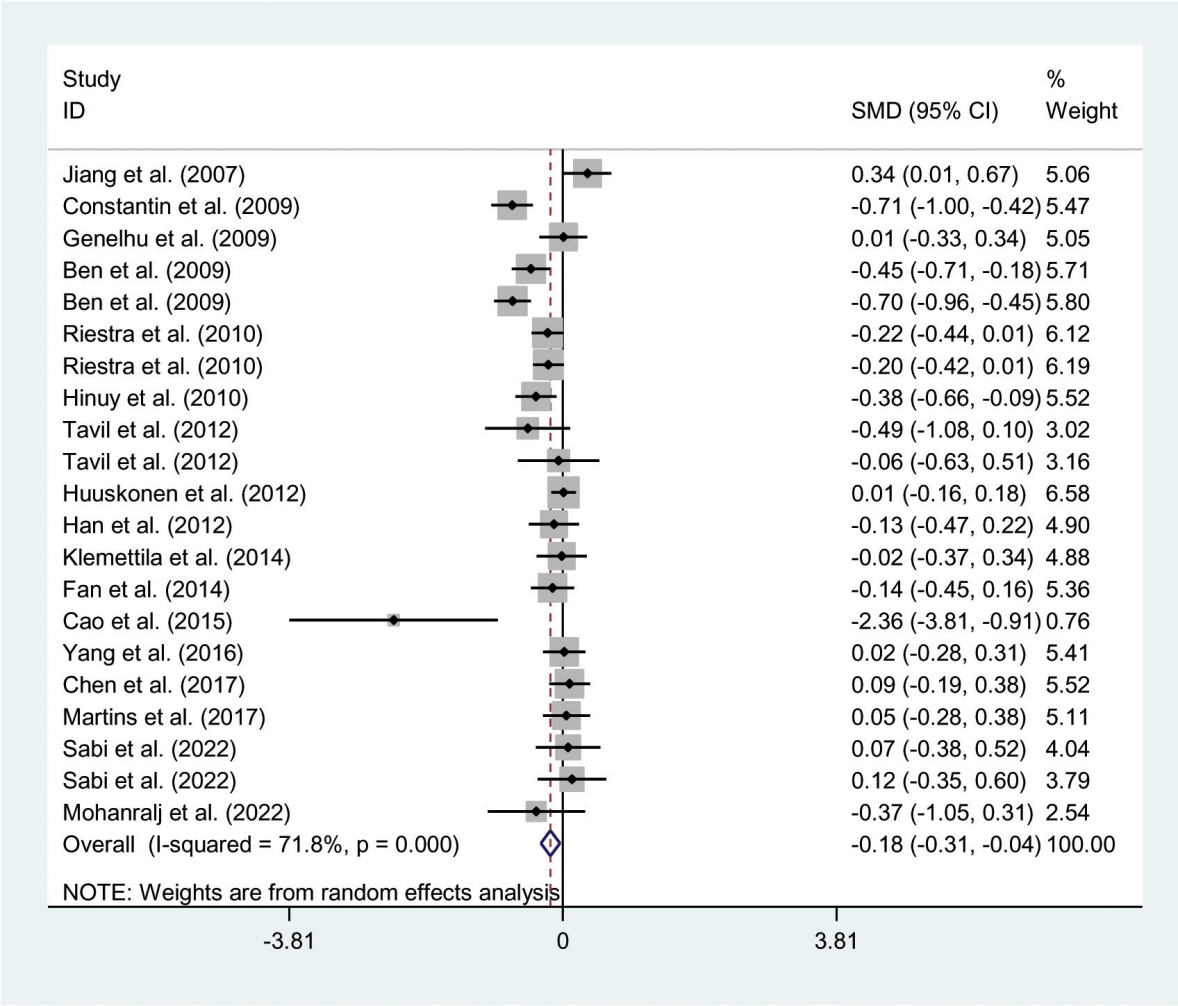
Forest plot of the association between *LEP* rs7799039 variant and leptin. *LEP*, leptin gene; SMD, standardized mean difference; 95% CI, 95% confidence interval.

**Figure 3:**
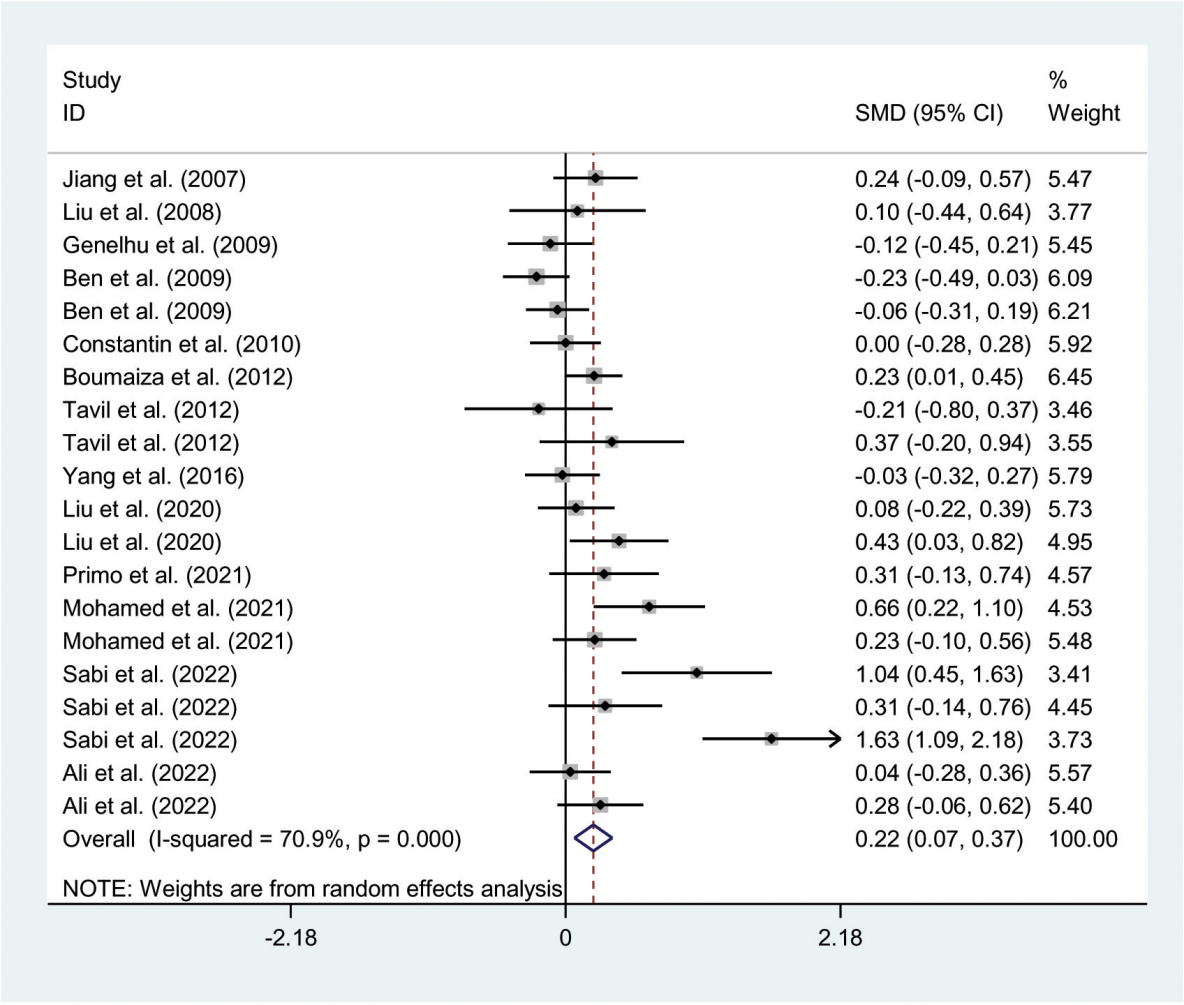
Forest plot of the association between *LEP* rs7799039 variant and insulin levels. *LEP*, leptin gene; SMD, standardized mean difference; 95% CI, 95% confidence interval.

**Figure 4:**
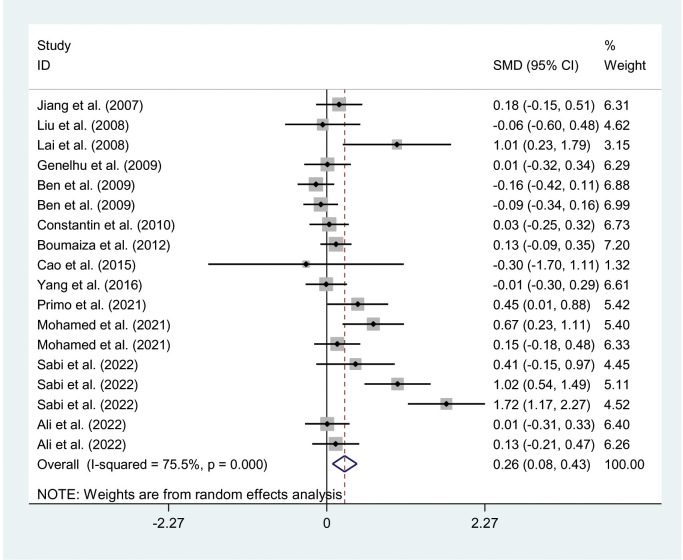
Forest plot of the association between *LEP* rs7799039 variant and HOMA-IR. *LEP*, leptin gene; SMD, standardized mean difference; 95% CI, 95% confidence; HOMA-IR, interval homeostasis model assessment of insulin resistance.

The relationships between the rs7739039 variant and leptin and glucose-lipid metabolism markers are presented in **Tables 1** and **2**. The pooled meta-analyses across the entire population revealed that carriers of the A-allele for the rs7739039 variant had lower fasting leptin levels (SMD = -0.18 ng/mL, 95% CI = -0.31 to -0.04 ng/mL, *p* = 0.01) (**Table 1**; **Figure 2**), and higher fasting insulin levels (SMD = 0.22 μmol/μL, 95% CI = 0.07 to 0.37 μmol/μL, *p* < 0.01) (**Table 1**; **Figure 3**), as well as higher HOMA-IR (SMD = 0.26, 95% CI = 0.08 to 0.43, *p* < 0.01) (**Table 1**; **Figure 4**), compared to the GG homozygotes. No significant associations were found between the rs7739039 variant and fasting levels of glucose, triglycerides, TC, LDL-C, or HDL-C in the overall population (**Supplementary Figures S1-S5**).

**Table 1:** Meta-analyses between *LEP* rs7799039 variant and leptin and glucose metabolism markers.

Groups or subgroups	Comparisons (Subjects)	SMD (95% CI)	*P*_Heterogeneity_	*P*_SMD_
Leptin (AA + AG vs. GG)				
All subjects	21 (5546)	-0.18 (-0.31, -0.04)	< 0.001	0.01
Males	3 (1339)	-0.09 (-0.24, 0.06)	0.29	0.24
Adults	16 (4496)	-0.18 (-0.35, -0.002)	< 0.001	0.047
Children/adolescents	5 (1050)	-0.18 (-0.31, -0.04)	0.70	0.01
Caucasians	5 (1823)	-0.22 (-0.45, 0.01)	0.001	0.06
Latin Americans	3 (494)	-0.12 (-0.40, 0.16)	0.10	0.40
Asians	11 (2749)	-0.05 (-0.23, 0.13)	0.03	0.60
Overweight/obesity patients	4 (525)	-0.10 (-0.40, 0.20)	0.05	0.52
General/control subjects	7 (1970)	-0.25 (-0.46, -0.03)	0.001	0.03
Glucose (AA + AG vs. GG)				
All subjects	24 (5486)	0.09 (-0.08, 0.27)	< 0.001	0.30
Adults	20 (4688)	0.15 (-0.06, 0.35)	< 0.001	0.17
Africans	7 (2253)	-0.07 (-0.24, 0.11)	0.01	0.45
Latin Americans	4(562)	-0.06 (-0.30, 0.17)	0.17	0.61
Asians	11 (2347)	0.38 (-0.01, 0.77)	< 0.001	0.06
T2DM patients	4 (671)	0.46 (-0.50, 1.42)	< 0.001	0.35
Overweight/obesity patients	7 (926)	0.69 (-0.03, 1.41)	< 0.001	0.06
General/control subjects	7 (2134)	-0.01 (-0.10, 0.09)	0.43	0.90
Insulin (AA + AG vs. GG)				
All subjects	20 (3811)	0.22 (0.07, 0.37)	< 0.001	< 0.01
Adults	16 (3440)	0.21 (0.04, 0.38)	< 0.001	0.01
Africans	5 (1038)	0.13 (-0.12, 0.39)	0.003	0.31
Asians	12 (2309)	0.32 (0.10, 0.55)	< 0.001	< 0.01
T2DM patients	4 (671)	0.50 (-0.10, 1.09)	<0.001	0.10
Overweight/obesity patients	6 (790)	0.32 (-0.11, 0.75)	<0.001	0.15
General/control subjects	5 (607)	0.26 (-0.13, 0.65)	0.001	0.19
HOMA-IR (AA + AG vs. GG)				
All subjects	18 (3455)	0.26 (0.08, 0.43)	< 0.001	< 0.01
Adults	16 (3226)	0.24 (0.05, 0.43)	< 0.001	0.01
Africans	5 (1038)	0.10 (-0.12, 0.32)	0.02	0.38
Asians	10 (1953)	0.42 (0.08, 0.75)	< 0.001	0.02
T2DM patients	3 (471)	0.59 (-0.42, 1.59)	<0.001	0.25
Overweight/obesity patients	6 (790)	0.50 (0.01, 0.98)	< 0.001	0.04
General/control subjects	4 (537)	0.20 (-0.14, 0.54)	0.02	0.24

*LEP*, leptin gene; SMD, standardized mean difference; 95% CI, 95% confidence interval; HOMA-IR, homeostasis model assessment of insulin resistance; T2DM, type 2 diabetes mellitus.

**Table 2:** Meta-analyses between *LEP* rs7799039 variant and lipid metabolism markers.

Groups or subgroups	Comparisons (Subjects)	SMD (95% CI)	*P*_Heterogeneity_	*P*_SMD_
Triglycerides (AA + AG vs. GG)				
All subjects	26 (4825)	-0.04 (-0.14, 0.05)	< 0.01	0.37
Adults	22 (4027)	-0.02 (-0.12, 0.08)	0.03	0.75
Asians	13 (2707)	-0.05 (-0.21, 0.12)	< 0.01	0.59
Latin Americans	4 (562)	-0.10 (-0.28, 0.07)	0.64	0.25
Africans	7 (1232)	-0.03 (-0.21, 0.16)	0.02	0.77
Overweight/obesity patients	7 (926)	0.13 (-0.03, 0.30)	0.21	0.12
T2DM patients	4 (889)	0.14 (-0.27, 0.55)	< 0.01	0.51
General/control subjects	5 (863)	-0.20 (-0.45, 0.06)	0.04	0.13
TC (AA + AG vs. GG)				
All subjects	26 (5540)	0.05 (-0.05, 0.15)	< 0.001	0.29
Adults	22 (4742)	0.06 (-0.04, 0.15)	0.02	0.25
Asians	12 (2301)	0.05 (-0.10, 0.20)	0.04	0.52
Latin Americans	4 (562)	-0.09 (-0.26, 0.09)	0.83	0.35
Africans	8 (2353)	0.12 (-0.09, 0.34)	< 0.001	0.26
Overweight/obesity patients	7 (926)	0.09 (-0.05, 0.22)	0.45	0.23
T2DM patients	3 (644)	0.01 (-0.29, 0.32)	0.07	0.93
General/control subjects	6 (1984)	0.02 (-0.08, 0.12)	0.54	0.68
LDL-C (AA + AG vs. GG)				
All subjects	26 (5456)	-0.03 (-0.09, 0.03)	0.70	0.34
Adults	22 (4658)	-0.05 (-0.12, 0.02)	0.93	0.13
Asians	13 (2546)	0.001 (-0.09, 0.09)	0.83	0.98
Latin Americans	4 (562)	-0.09 (-0.27, 0.08)	0.75	0.30
Africans	7 (2024)	-0.01 (-0.11, 0.10)	0.37	0.90
Overweight/obesity patients	7 (926)	-0.01 (-0.20, 0.17)	0.12	0.88
T2DM patients	4 (889)	-0.05 (-0.21, 0.11)	0.49	0.55
General/control subjects	6 (1984)	-0.02 (-0.12, 0.09)	0.87	0.77
HDL-C (AA + AG vs. GG)				
All subjects	27 (5785)	0.10 (-0.05, 0.25)	< 0.001	0.20
Adults	23 (4987)	0.12 (-0.04, 0.27)	< 0.001	0.15
Asians	13 (2546)	-0.02 (-0.17, 0.14)	0.01	0.86
Latin Americans	4 (562)	0.13 (-0.04, 0.31)	0.85	0.14
Africans	8 (2353)	0.18 (-0.16, 0.50)	< 0.001	0.30
Overweight/obesity patients	7 (926)	0.05 (-0.13, 0.23)	0.14	0.56
T2DM patients	4 (889)	0.05 (-0.31, 0.42)	0.01	0.78
General/control subjects	6 (1984)	0.22 (-0.14, 0.59)	< 0.001	0.23

*LEP*, leptin gene; SMD, standardized mean difference; 95% CI, 95% confidence interval; TC, total cholesterol; LDL-C, low-density lipoprotein cholesterol; HDL-C, high-density lipoprotein cholesterol; T2DM, type 2 diabetes mellitus.

The interactions between the rs7739039 variant and ethnicity and health condition on leptin and glucose metabolism markers were observed (**Table 1**). The A allele of the rs7739039 variant is linked to increased insulin (SMD = 0.32 μmol/μL, 95% CI = 0.10 to 0.55 μmol/μL, *p* < 0.01) and HOMA-IR (SMD = 0.42, 95% CI = 0.08 to 0.75, *p* = 0.02) levels in Asians, but not in Africans. The A allele carriers have a greater HOMA-IR (SMD = 0.50, 95% CI = 0.01 to 0.98, *p* = 0.04) than the GG homozygotes in overweight/obesity patients, but not in individuals with T2DM. Additionally, the A allele of the rs7739039 variant is associated with reduced leptin levels in general/control subjects (SMD = -0.25 ng/mL, 95% CI = -0.46 to -0.03 ng/mL, *p* = 0.03), but not in overweight/obesity patients. No significant associations between the rs7739039 variant and any of the lipid metabolism markers were detected in the pooled meta-analyses of the whole population (**Table 2**).

### Associations of *LEPR* rs1137100, rs1137101 and rs1805094 variants with leptin and glucose-lipid metabolism markers

No significant associations were found between *LEPR* rs1137100 (**Supplementary Tables S14**, **S15**; **Supplementary Figures S6-S13**), rs1137101 (**Tables 3**, **4**; **Supplementary Figures S14-S21**), and rs1805094 (**Supplementary Tables S16**, **S17**; **Supplementary Figures S22-S29**) variants and leptin levels or any markers of glucose-lipid metabolism across the entire population. However, subgroup analyses revealed significant associations between the G allele of the rs1137101 variant and lower leptin levels (SMD = -0.16 ng/mL, 95% CI = -0.33 to -0.001 ng/mL, *p* = 0.049) in Africans, as well as higher glucose levels (SMD = 0.17 mg/dL, 95% CI = 0.001 to 0.33 mg/dL, *p* = 0.049) in individuals with overweight/obesity (**Table 3**). Carriers of the G allele of rs1137101 also exhibited higher TC levels (SMD = 0.15 mg/dL, 95% CI = 0.01 to 0.29 mg/dL, *p* = 0.03) in the general/control subjects, but lower LDL-C levels (SMD = -0.34 mg/dL, 95% CI = -0.65 to -0.02 mg/dL, *p* = 0.04) in Asians compared to those with the AA genotype (**Table 4**). Furthermore, C-allele carriers of the rs1805094 variant showed elevated leptin levels in males (SMD = 0.29 ng/mL, 95% CI = 0.07 to 0.51 ng/mL, *p* < 0.01), children/adolescents (SMD = 0.17 ng/mL, 95% CI = 0.06 to 0.28 ng/mL, *p* < 0.01), and Caucasians (SMD = 0.20 ng/mL, 95% CI = 0.06 to 0.33 ng/mL, *p* < 0.01) than the GG homozygotes (**Supplementary Table S16**). Somehow, they had lower leptin levels than the GG homozygotes in Asians (**Supplementary Table S16**). C-allele carriers of the rs1805094 variant also had lower triglyceride levels (SMD = -0.17 mg/dL, 95% CI = -0.31 to -0.03 mg/dL, *p* = 0.02) in overweight/obesity patients, lower LDL-C levels in Asians (SMD = -0.43 mg/dL, 95% CI = -0.81 to -0.06 mg/dL, *p* = 0.03), and lower HDL-C levels in males (SMD = -0.33 mg/dL, 95% CI = -0.56 to -0.09 mg/dL, *p* < 0.01) and in Caucasians (SMD = -0.14 mg/dL, 95% CI = -0.28 to -0.01 mg/dL, *p* = 0.04) compared to those with the GG genotype (**Supplementary Tables S16**, **S17**). No significant relationships were observed between the rs1137100 variant and leptin or glucose-lipid metabolism markers in both the overall and subgroup analyses (**Supplementary Tables S14**, **S15**).

**Table 3:** Meta-analyses between *LEPR* rs1137101 variant and leptin and glucose metabolism markers.

Groups or subgroups	Comparisons (subjects)	SMD (95% CI)	*P*_Heterogeneity_	*P*_SMD_
Leptin (GG + AG vs. AA)				
All subjects	43 (11391)	0.05 (-0.04, 0.14)	< 0.001	0.24
Females	12 (1719)	0.12 (-0.03, 0.28)	0.04	0.11
Males	9 (2048)	0.27 (-0.06, 0.59)	< 0.001	0.11
Adults	37 (9322)	0.09 (-0.02, 0.20)	< 0.001	0.09
Children/adolescents	6 (2069)	-0.07 (-0.18, 0.03)	0.33	0.15
Caucasians	23 (6112)	0.07 (-0.05, 0.19)	< 0.001	0.23
Latin Americans	6 (635)	-0.04 (-0.25, 0.17)	0.19	0.69
Asians	11 (3841)	0.13 (-0.10, 0.35)	< 0.01	0.27
Africans	3 (803)	-0.16 (-0.33, -0.001)	0.30	0.049
Overweight/obesity patients	10 (1340)	0.21 (-0.12, 0.54)	< 0.001	0.21
General/control subjects	24 (5283)	0.03 (-0.06, 0.12)	0.01	0.51
Glucose (GG + AG vs. AA)				
All subjects	33 (9865)	-0.01 (-0.10, 0.08)	< 0.001	0.85
Females	7 (570)	0.07 (-0.11, 0.24)	0.57	0.46
Adults	31 (9424)	-0.02 (-0.11, 0.08)	< 0.001	0.72
Caucasians	11 (2165)	-0.01 (-0.14, 0.12)	0.13	0.85
Latin Americans	3 (538)	-0.03 (-0.46, 0.39)	0.01	0.88
Asians	16 (5602)	-0.05 (-0.22, 0.13)	< 0.001	0.60
Africans	3 (1560)	0.08 (-0.19, 0.35)	0.01	0.56
Overweight/obesity patients	7 (648)	0.17 (0.001, 0.33)	0.81	0.049
General/control subjects	13 (4050)	-0.04 (-0.17, 0.08)	0.01	0.50
Insulin (GG + AG vs. AA)				
All subjects	23 (5584)	0.06 (-0.07, 0.19)	< 0.001	0.34
Males	3 (415)	-0.13 (-0.47, 0.21)	0.16	0.44
Females	7 (570)	0.12 (-0.22, 0.46)	< 0.01	0.50
Adults	22 (5481)	0.07 (-0.06, 0.21)	< 0.001	0.28
Caucasians	11 (1666)	-0.02 (-0.18, 0.14)	0.05	0.80
Latin Americans	3 (332)	0.02 (-0.26, 0.29)	0.24	0.90
Asians	7 (3147)	0.15 (-0.17, 0.48)	< 0.001	0.36
Overweight/obesity patients	6 (538)	0.18 (-0.16, 0.51)	0.02	0.30
General/control subjects	8 (907)	-0.01 (-0.20, 0.18)	0.14	0.94
HOMA-IR (GG + AG vs. AA)				
All subjects	17 (5301)	0.06 (-0.12, 0.24)	< 0.001	0.53
Females	5 (485)	0.21 (-0.14, 0.55)	0.02	0.24
Caucasians	5 (1418)	0.03 (-0.09, 0.14)	0.40	0.66
Asians	9 (3347)	0.06 (-0.34, 0.45)	< 0.001	0.78
General/control subjects	6 (983)	0.01 (-0.16, 0.19)	0.19	0.87

*LEP*, leptin receptor gene; SMD, standardized mean difference; 95% CI, 95% confidence interval; HOMA-IR, homeostasis model assessment of insulin resistance.

**Table 4:** Meta-analyses between *LEP* rs1137101 variant and lipid-metabolism markers.

Groups or subgroups	Comparisons (subjects)	SMD (95% CI)	*P*_Heterogeneity_	*P*_SMD_
Triglycerides (GG + AG vs. AA)				
All subjects	33 (9920)	0.04 (-0.06, 0.14)	< 0.001	0.44
Males	4 (614)	-0.18 (-0.39, 0.03)	0.23	0.10
Females	6 (565)	0.15 (-0.02, 0.32)	0.85	0.09
Adults	32 (9792)	0.03 (-0.07, 0.14)	< 0.001	0.52
Caucasians	6 (1563)	0.03 (-0.24, 0.30)	< 0.001	0.83
Asians	21 (7000)	0.02 (-0.12, 0.15)	< 0.001	0.80
Africans	4 (1132)	0.05 (-0.16, 0.26)	0.03	0.63
Overweight/obesity patients	9 (1493)	0.04 (-0.07, 0.15)	0.98	0.43
T2DM patients	3 (632)	0.06 (-0.10, 0.22)	0.71	0.44
Hypertensive patients	3 (446)	0.003 (-0.23, 0.23)	0.88	0.98
General/control subjects	13 (3672)	0.08 (-0.07, 0.23)	0.001	0.28
TC (GG + AG vs. AA)				
All subjects	35 (10716)	-0.04 (-0.21, 0.13)	< 0.001	0.68
Males	5 (736)	-0.07 (-0.22, 0.09)	0.75	0.40
Females	7 (674)	-0.67 (-1.87, 0.52)	< 0.001	0.27
Adults	34 (10588)	-0.04 (-0.21, 0.14)	< 0.001	0.69
Caucasians	8 (1794)	0.06 (-0.10, 0.23)	0.06	0.44
Asians	20 (6444)	-0.17 (-0.49, 0.15)	< 0.001	0.29
Africans	5 (2253)	0.06 (-0.09, 0.20)	0.06	0.44
Overweight/obesity patients	9 (1493)	0.12 (-0.07, 0.30)	0.01	0.21
T2DM patients	3 (632)	0.04 (-0.12, 0.19)	0.80	0.66
Hypertensive patients	3 (446)	0.07 (-0.22, 0.36)	0.25	0.64
General/control subjects	15 (4468)	0.15 (0.01, 0.29)	< 0.001	0.03
LDL-C (GG + AG vs. AA)				
All subjects	33 (10919)	-0.17 (-0.35, 0.01)	< 0.001	0.07
Males	4 (712)	-0.09 (-0.25, 0.07)	1.00	0.26
Females	7 (674)	0.05 (-0.11, 0.21)	0.99	0.52
Adults	32 (10791)	-0.17 (-0.36, 0.01)	< 0.001	0.07
Caucasians	7 (2326)	0.06 (-0.10, 0.22)	0.06	0.44
Asians	20 (6444)	-0.34 (-0.65, -0.02)	< 0.001	0.04
Africans	4 (1924)	0.01 (-0.18, 0.19)	0.03	0.94
Overweight/obesity patients	9 (1493)	-0.004 (-0.11, 0.10)	< 0.001	0.94
T2DM patients	3 (632)	0.06 (-0.10, 0.21)	0.46	0.48
Hypertensive patients	3 (446)	-0.12 (-0.35, 0.11)	0.40	0.31
General/control subjects	13 (3966)	-0.16 (-0.51, 0.19)	1.00	0.37
HDL-C (GG + AG vs. AA)				
All subjects	36 (11272)	-0.02 (-0.12, 0.09)	< 0.001	0.76
Males	5 (736)	-0.12 (-0.74, 0.49)	< 0.001	0.70
Females	7 (674)	-0.10 (-0.26, 0.06)	1.00	0.20
Adults	35 (11144)	-0.02 (-0.12, 0.09)	< 0.001	0.76
Caucasians	8 (1794)	-0.04 (-0.33, 0.26)	< 0.001	0.81
Asians	21 (7000)	-0.02 (-0.16, 0.13)	< 0.001	0.82
Africans	5 (2253)	-0.01 (-0.26, 0.24)	< 0.001	0.93
Overweight/obesity patients	9 (1493)	-0.11 (-0.37, 0.16)	< 0.001	0.43
T2DM patients	3 (632)	-0.27 (-0.61, 0.06)	0.03	0.11
Hypertensive patients	3 (446)	-0.34 (-0.70, 0.01)	0.14	0.06
General/control subjects	16 (5024)	-0.02 (-0.14, 0.10)	< 0.001	0.77

*LEP*, leptin receptor gene; SMD, standardized mean difference; 95% CI, 95% confidence interval; TC, total cholesterol; LDL-C, low-density lipoprotein cholesterol; HDL-C, high-density lipoprotein cholesterol; T2DM, type 2 diabetes mellitus.

### Heterogeneity analysis

Significant heterogeneity was observed in the pooled meta-analyses for leptin, glucose, insulin, HOMA-IR, triglycerides, TC, and HDL-C for the rs7799039 variant in the entire population (**Tables 1**, **2**). Similarly, significant heterogeneity was found in the pooled meta-analyses for leptin, glucose, insulin, HOMA-IR, triglycerides, TC, LDL-C, and HDL-C for the rs1137101 variant across the whole population (**Tables 3**, **4**). For the rs1805094 variant, significant heterogeneity was noted in the pooled meta-analyses for leptin, glucose, insulin, HOMA-IR, and HDL-C in the entire population (**Supplementary Tables S16**, **S17**). Additionally, significant heterogeneity was present for leptin, TC, LDL-C, and HDL-C in the pooled meta-analyses for the rs1137100 variant in the whole population (**Supplementary Tables S14**, **S15**). The sources of heterogeneity were effectively identified using Galbraith plots. Excluding outlier studies resulted in a significant reduction or elimination of the heterogeneity, while the results of the pooled meta-analyses remained largely unchanged for all indexes.

### Publication bias

Publication bias was detected in the association analyses between the rs7799039 variant and insulin levels as well as HOMA-IR. To correct for this bias, the trim-and-fill method was applied, and the adjusted pooled results showed no significant changes for either marker after the adjustment.

## Discussion

Leptin and its receptor are key components in regulating energy homeostasis, and any disruption in their signaling pathway can lead to the development or exacerbation of the metabolic diseases such as obesity, T2DM, metabolic syndrome, non-alcoholic fatty liver disease, and CVD (38–40). Several studies have suggested that the rs7799039 variant in *LEP*, as well as the rs1137100, rs1137101, and rs1805094 variants in *LEPR*, is linked to the onset of T2DM and CVD (41–43). This led us to conduct the present meta-analysis to clarify the relationships between these four polymorphic loci and glucose-lipid metabolism markers including glucose, insulin, HOMA-IR, triglycerides, TC, LDL-C and HDL-C. Our meta-analysis reveals that the A allele of the rs7799039 variant in the *LEP* promoter is associated with lower leptin levels but higher insulin and HOMA-IR levels. This aligns with previous studies indicating that the A allele of rs7799039 is strongly linked to an increased risk of T2DM (41, 43). Furthermore, we found that *LEPR* rs1137101 and rs1805094 variants are significantly associated with leptin, glucose, and cholesterol levels in specific populations, such as Africans and individuals with overweight/obesity, although these variants were not associated with glucose-lipid metabolism markers in the pooled meta-analyses in the overall population.

The mechanisms behind the associations of the rs7799039 variant with leptin and glucose metabolism markers could be attributed to the A allele of this variant, which leads to reduced *LEP* mRNA expression due to its location in the gene’s promoter region. However, Kolić et al. (44) examined the levels of *LEP* mRNA in peripheral blood mononuclear cells of patients with relapsing-remitting multiple sclerosis who carried different rs7799039 polymorphic genotypes. They found that *LEP* mRNA levels in the A allele carriers were significantly higher than in patients with the GG genotype. We utilized GTEx Analysis Release V8 (dbGaP Accession phs000424.v8.p2) to examine the effect of the rs7799039 variant on *LEP* mRNA expression. The analysis displayed that the A allele of the rs7799039 variant is significantly associated with higher levels of *LEP* mRNA in both cultured fibroblasts and tibial arteries (*p* < 0.00001 for both). We speculate that the impact of rs7799039 on leptin gene expression is tissue-specific, with its regulatory effect on leptin gene expression varying across different tissues. Of course, this remains merely a hypothesis, as there is currently no further evidence to support this. However, when we examined the influence of the rs1137101 variant on *LEP* mRNA levels using GTEx Analysis Release V8 (dbGaP Accession phs000424.v8.p2), we found that the G allele was significantly negatively correlated with *LEP* mRNA levels in cultured fibroblasts and the tibial artery, while it showed a significant positive correlation in tissues such as subcutaneous adipose, brain, and thyroid. This indirectly supports the possibility that the effect of rs7799039 variant on leptin gene expression could be tissue-specific.

In this study, *LEPR* variants rs1137101 and rs1805094 exhibited a weak association with glucose-lipid metabolic markers, while the rs1137100 variant showed no significant association with any of these markers. This disparity may be attributed to the distinct properties of the substituted amino acids. The rs1137100, rs1137101, and rs1805094 variants are all missense mutations, and the functional impact of these mutations on LEPR depends on the differences in properties between the substituted and original amino acids. The greater the differences in properties, the more pronounced the effect on the structure, stability, and function of the protein. An example of a missense mutation with significant clinical implications is the mutation responsible for sickle cell anemia, which occurs in the hemoglobin β-globin gene (45). In this condition, the sixth glutamic acid in the β-chain is substituted by valine. Glutamic acid is a polar acidic amino acid typically located on the surface of the protein, while valine is a nonpolar, neutral amino acid that is generally found in the protein’s interior. This substitution alters hemoglobin’s structure, transforming it from a soluble globular form to an insoluble, tubular, or fibrous polymer. As a result, red blood cells assume a rigid sickle shape, impairing their ability to carry oxygen. In contrast, the rs1137100 variant involves the substitution of lysine with arginine. Both amino acids are polar basic residues, which suggests that this substitution has minimal effect on the molecular structure and function of LEPR, and thus no significant impact on glucose and lipid metabolism. The rs1137101 variant involves the substitution of glutamine with arginine, and the rs1805094 variant involves the substitution of lysine with asparagine. Although all of these amino acids are polar, arginine and lysine are polar basic amino acids, while glutamine and asparagine are polar neutral amino acids, indicating some difference in their properties. Therefore, the rs1137101 and rs1805094 variants may modestly affect the structure and function of LEPR, leading to a weak association with glucose-lipid metabolism markers observed in this study.

Significant heterogeneity was observed in the pooled meta-analyses for the total population regarding the associations between the rs7799039 variant and leptin, insulin, or HOMA-IR. Outlier studies were identified using Galbraith plots, and exclusion of these studies did not result in significant changes to SMDs or 95% CIs. This suggests that the associations between the rs7799039 variant and these markers are robust. Similarly, significant heterogeneity was detected in the association meta-analyses for the rs1137100, rs1137101, and rs1805094 variants with glucose-lipid metabolism markers in the total population. After identifying and excluding outlier studies, no significant correlations remained, indicating that the associations between these variants and glucose-lipid metabolic markers are weak.

This study has several limitations. First, only studies published in English and Chinese were included in this meta-analysis due to challenges in accessing full-text articles from studies published in other languages. Second, subgroup analyses were limited to age, gender, ethnicity, and health condition. The interactions between the rs7799039, rs1137100, rs1137101, and rs1805094 variants and other genetic or non-genetic factors influencing glucose-lipid metabolism markers were not explored due to the lack of original data from the included studies. It is well established that numerous factors, such as additional genetic variations, diet, physical activity, and environmental influences, contribute to glycolipid metabolic disorders (46–48). More comprehensive insights could be gained if gene-gene interactions were examined or if stratification analyses incorporating factors like diet, physical activity, and environmental exposures were performed.

In conclusion, the A allele of the rs7799039 variant in the promoter of *LEP* is associated with lower levels of leptin, and confers a higher risk of insulin resistance. *LEPR* rs1137101, and rs1805094 variants are weakly correlated with glucose-lipid metabolism markers, while *LEPR* rs1137100 variant is not associated with any of the glucose-lipid metabolism markers. These findings may partially help explain the interrelationships among these genetic variants, leptin levels, metabolic disorders and the development of diabetes and cardiovascular diseases.

## Data Availability

The original contributions presented in the study are included in the article/**Supplementary Material**. Further inquiries can be directed to the corresponding author.
