# Supplementary material for: The rs7799039 variant in the leptin gene promoter drives insulin resistance through reduced serum leptin levels
Source: Front Endocrinol (Lausanne). 2025 Oct 13;16:1589575. doi: 10.3389/fendo.2025.1589575 (PMC12554595; doi:10.3389/fendo.2025.1589575)
Supplement: Supplementary file 1 [file DataSheet1.docx]

**Supplementary Figures S1-S29**

| **Figure S1** | Forest plot of the association between *LEP* rs7799039 variant and glucose levels. |
| --- | --- |
| **Figure S2** | Forest plot of the association between *LEP* rs7799039 variant and triglyceride levels. |
| **Figure S3** | Forest plot of the association between *LEP* rs7799039 variant and total cholesterol (TC) levels. |
| **Figure S4** | Forest plot of the association between *LEP* rs7799039 variant and low-density lipoprotein cholesterol (LDL-C) levels. |
| **Figure S5** | Forest plot of the association between *LEP* rs7799039 variant and high-density lipoprotein cholesterol (HDL-C) levels. |
| **Figure S6** | Forest plot of the association between *LEPR* rs1137100 variant and leptin levels. |
| **Figure S7** | Forest plot of the association between *LEPR* rs1137100 variant and glucose levels. |
| **Figure S8** | Forest plot of the association between *LEPR* rs1137100 variant and insulin levels. |
| **Figure S9** | Forest plot of the association between *LEPR* rs1137100 variant and homeostasis model assessment of insulin resistance (HOMA-IR). |
| **Figure S10** | Forest plot of the association between *LEPR* rs1137100 variant and triglyceride levels. |
| **Figure S11** | Forest plot of the association between *LEPR* rs1137100 variant and total cholesterol (TC) levels. |
| **Figure S12** | Forest plot of the association between *LEPR* rs1137100 variant and low-density lipoprotein cholesterol (LDL-C) levels. |
| **Figure S13** | Forest plot of the association between *LEPR* rs1137100 variant and high-density lipoprotein cholesterol (HDL-C) levels. |
| **Figure S14** | Forest plot of the association between *LEPR* rs1137101 variant and leptin levels. |
| **Figure S15** | Forest plot of the association between *LEPR* rs1137101 variant and glucose levels. |
| **Figure S16** | Forest plot of the association between *LEPR* rs1137101 variant and insulin levels. |
| **Figure S17** | Forest plot of the association between *LEPR* rs1137101 variant and homeostasis model assessment of insulin resistance (HOMA-IR). |
| **Figure S18** | Forest plot of the association between *LEPR* rs1137101 variant and triglyceride levels. |
| **Figure S19** | Forest plot of the association between *LEPR* rs1137101 variant and total cholesterol (TC) levels. |
| **Figure S20** | Forest plot of the association between *LEPR* rs1137101 variant and low-density lipoprotein cholesterol (LDL-C) levels. |
| **Figure S21** | Forest plot of the association between *LEPR* rs1137101 variant and high-density lipoprotein cholesterol (HDL-C) levels. |
| **Figure S22** | Forest plot of the association between LEPR rs1805094 variant and leptin levels. |
| **Figure S23** | Forest plot of the association between *LEPR* rs1805094 variant and glucose levels. |
| **Figure S24** | Forest plot of the association between *LEPR* rs1805094 variant and insulin levels. |
| **Figure S25** | Forest plot of the association between *LEPR* rs1805094 variant and homeostasis model assessment of insulin resistance (HOMA-IR). |
| **Figure S26** | Forest plot of the association between *LEPR* rs1805094 variant and triglyceride levels. |
| **Figure S27** | Forest plot of the association between *LEPR* rs1805094 variant and total cholesterol (TC) levels. |
| **Figure S28** | Forest plot of the association between *LEPR* rs1805094 variant and low-density lipoprotein cholesterol (LDL-C) levels. |
| **Figure S29** | Forest plot of the association between *LEPR* rs1805094 variant and high-density lipoprotein cholesterol (HDL-C) levels. |

**Figure S1.** Forest plot of the association between *LEP* rs7799039 variant and glucose levels.

**Figure S2.** Forest plot of the association between *LEP* rs7799039 variant and triglyceride levels.

**Figure S3.** Forest plot of the association between *LEP* rs7799039 variant and total cholesterol (TC) levels.

**Figure S4.** Forest plot of the association between *LEP* rs7799039 variant and low-density lipoprotein cholesterol (LDL-C) levels.

**Figure S5.** Forest plot of the association between *LEP* rs7799039 variant and high-density lipoprotein cholesterol (HDL-C) levels.

**Figure S6.** Forest plot of the association between *LEPR* rs1137100 variant and leptin levels.

**Figure S7.** Forest plot of the association between *LEPR* rs1137100 variant and glucose levels.

**Figure S8.** Forest plot of the association between *LEPR* rs1137100 variant and insulin levels.

**Figure S9.** Forest plot of the association between *LEPR* rs1137100 variant and homeostasis model assessment of insulin resistance (HOMA-IR).

**Figure S10.** Forest plot of the association between *LEPR* rs1137100 variant and triglyceride levels.

**Figure S11.** Forest plot of the association between *LEPR* rs1137100 variant and total cholesterol (TC) levels.

**Figure S12.** Forest plot of the association between *LEPR* rs1137100 variant and low-density lipoprotein cholesterol (LDL-C) levels.

**Figure S13.** Forest plot of the association between *LEPR* rs1137100 variant and high-density lipoprotein cholesterol (HDL-C) levels.

**Figure S14.** Forest plot of the association between *LEPR* rs1137101 variant and leptin levels.

**Figure S15.** Forest plot of the association between *LEPR* rs1137101 variant and glucose levels.

**Figure S16.** Forest plot of the association between *LEPR* rs1137101 variant and insulin levels.

**Figure S17.** Forest plot of the association between *LEPR* rs1137101 variant and homeostasis model assessment of insulin resistance (HOMA-IR).

**Figure S18.** Forest plot of the association between *LEPR* rs1137101 variant and triglyceride levels.

**Figure S19.** Forest plot of the association between *LEPR* rs1137101 variant and total cholesterol (TC) levels.

**Figure S20.** Forest plot of the association between *LEPR* rs1137101 variant and low-density lipoprotein cholesterol (LDL-C) levels.

**Figure S21.** Forest plot of the association between *LEPR* rs1137101 variant and high-density lipoprotein cholesterol (HDL-C) levels.

**Figure S22.** Forest plot of the association between *LEPR* rs1805094 variant and leptin levels.

**Figure S23.** Forest plot of the association between *LEPR* rs1805094 variant and glucose levels.

**Figure S24.** Forest plot of the association between *LEPR* rs1805094 variant and insulin levels.

**Figure S25.** Forest plot of the association between *LEPR* rs1805094 variant and homeostasis model assessment of insulin resistance (HOMA-IR).

**Figure S26.** Forest plot of the association between *LEPR* rs1805094 variant and triglyceride levels.

**Figure S27.** Forest plot of the association between *LEPR* rs1805094 variant and total cholesterol (TC) levels.

**Figure S28.** Forest plot of the association between *LEPR* rs1805094 variant and low-density lipoprotein cholesterol (LDL-C) levels.

**Figure S29.** Forest plot of the association between *LEPR* rs1805094 variant and high-density lipoprotein cholesterol (HDL-C) levels.
