# Supplementary material for: The rs7799039 variant in the leptin gene promoter drives insulin resistance through reduced serum leptin levels
Source: Front Endocrinol (Lausanne). 2025 Oct 13;16:1589575. doi: 10.3389/fendo.2025.1589575 (PMC12554595; doi:10.3389/fendo.2025.1589575)
Supplement: Supplementary file 2 [file DataSheet2.docx]

**Supplementary Tables S1-S17**

**Table S1.** Reference list of the studies included in the meta-analysis.

**Table S2.** Characteristics of the studies included in the meta-analysis for *LEP* rs7799039 variant.

**Table S3.** Original data for leptin and glucose metabolism markers by the genotypes of *LEP* rs7799039 variant.

**Table S4.** Original data for lipid metabolism markers by the genotypes of *LEP* rs7799039 variant.

**Table S5.** Characteristics of the studies included in the meta-analysis for *LEPR* rs1137100 variant.

**Table S6.** Original data for leptin and glucose metabolism markers by the genotypes of *LEPR* rs1137100 variant.

**Table S7.** Original data for lipid metabolism markers by the genotypes of *LEPR* rs1137100 variant.

**Table S8.** Characteristics of the studies included in the meta-analysis for *LEPR* rs1137101 variant.

**Table S9.** Original data for leptin and glucose metabolism markers by the genotypes of *LEPR* rs1137101 variant.

**Table S10.** Original data for lipid metabolism markers by the genotypes of *LEPR* rs1137101 variant.

**Table S11.** Characteristics of the studies included in the meta-analysis for *LEPR* rs1805094 variant.

**Table S12.** Original data for leptin and glucose metabolism markers by the genotypes of *LEPR* rs1805094 variant.

**Table S13.** Original data for lipid metabolism markers by the genotypes of *LEPR* rs1805094 variant.

**Table S14.** Meta-analyses between *LEPR* rs1137100 variant and leptin and glucose metabolism markers.

**Table S15.** Meta-analyses between *LEPR* rs1137100 variant and lipid-metabolism markers.

**Table S16.** Meta-analyses between *LEPR* rs1805094 variant and leptin and glucose metabolism markers.

**Table S17.** Meta-analyses between *LEPR* rs1805094 variant and lipid-metabolism markers.

**Table S1. Reference list of the studies included in the meta-analysis.**

| **NO** | Studies included for *LEP* rs7799039 variant |
| --- | --- |
| [1] | Jiang Y, Yao H, Sun YP et al. Study onthe associationof leptingene polymorphisms G2548A with hyperuricemiaamong males and analysis. Journal of Xinjiang Medical University. 2007; (06): 547-50. doi: 10.3969/j.issn.1009-5551.2007.06.004. |
| [2] | Liu HL, Lin YG, Wu J et al. Impact of genetic polymorphisms of leptin and TNF-alpha on rosiglitazone response in Chinese patients with type 2 diabetes. Eur J Clin Pharmacol. 2008; 64(7): 663-71. doi: 10.1007/s00228-008-0483-9. |
| [3] | Lai XB, Nie YQ, Li YL et al. The relationship between leptin gene G2548A polymorphism and insulin resistance in patients with non-alcoholic fatty liver disease: a report of 107 cases. New medicine. 2008; 39(12): 781-2. doi: 10.3969/j.issn.0253-9802.2008.12.005. |
| [4] | Ben Ali S, Kallel A, Ftouhi B et al. Association of G-2548A LEP polymorphism with plasma leptin levels in Tunisian obese patients. Clin Biochem. 2009; 42(7-8): 584-8. doi: 10.1016/j.clinbiochem.2008.11.001. |
| [5] | Genelhu VA, Celoria BM, Pimentel MM et al. Association of a common variant of the leptin gene with blood pressure in an obese Brazilian population. Am J Hypertens. 2009; 22(5): 577-80. doi: 10.1038/ajh.2009.7. |
| [6] | Constantin A, Costache G, Sima AV et al. Leptin G-2548A and leptin receptor Q223R gene polymorphisms are not associated with obesity in Romanian subjects. Biochem Biophys Res Commun. 2010; 391(1): 282-6. doi: 10.1016/j.bbrc.2009.11.050. |
| [7] | Hinuy HM, Hirata MH, Sampaio MF et al. Relationship between variants of the leptin gene and obesity and metabolic biomarkers in Brazilian individuals. Arq Bras Endocrinol Metabol. 2010; 54(3): 282-8. doi: 10.1590/s0004-27302010000300006. |
| [8] | Riestra P, Garcia-Anguita A, Viturro E et al. Influence of the leptin G-2548A polymorphism on leptin levels and anthropometric measurements in healthy Spanish adolescents. Ann Hum Genet. 2010; 74(4): 335-9. doi: 10.1111/j.1469-1809.2010.00586.x. |
| [9] | Abdel Hay RM, Rashed LA. Association between the leptin gene 2548G/A polymorphism, the plasma leptin and the metabolic syndrome with psoriasis. Exp Dermatol. 2011; 20(9): 715-9. doi: 10.1111/j.1600-0625.2011.01299.x. |
| [10] | Boumaiza I, Omezzine A, Rejeb J et al. Relationship between leptin G2548A and leptin receptor Q223R gene polymorphisms and obesity and metabolic syndrome risk in Tunisian volunteers. Genet Test Mol Biomarkers. 2012; 16(7): 726-33. doi: 10.1089/gtmb.2011.0324. |
| [11] | Han LH, Liu YQ. Exploration of the relationship between leptin gene 2548G/A polymorphism and susceptibility to obstructive sleep apnea hypopnea syndrome. shandong medical journal. 2012; 52(47): 64-6. doi: CNKI:SUN:SDYY.0.2012-47-029. |
| [12] | Huuskonen A, Lappalainen J, Oksala N et al. Aerobic fitness does not modify the effect of FTO variation on body composition traits. PLoS One. 2012; 7(12): e51635. doi: 10.1371/journal.pone.0051635. |
| [13] | Tavil B, Balta G, Ergun EL et al. Leptin promoter G-2548A genotypes and associated serum leptin levels in childhood acute leukemia at diagnosis and under high-dose steroid therapy. Leuk Lymphoma. 2012; 53(4): 648-53. doi: 10.3109/10428194.2011.626881. |
| [14] | Al-Azzam SI, Khabour OF, Alzoubi KH et al. The effect of leptin promoter and leptin receptor gene polymorphisms on lipid profile among the diabetic population: modulations by atorvastatin treatment and environmental factors. J Endocrinol Invest. 2014; 37(9): 835-42. doi: 10.1007/s40618-014-0113-6. |
| [15] | Fan SH, Say YH. Leptin and leptin receptor gene polymorphisms and their association with plasma leptin levels and obesity in a multi-ethnic Malaysian suburban population. J Physiol Anthropol. 2014; 33(1): 15. doi: 10.1186/1880-6805-33-15. |
| [16] | Ferreira-Julio MA, Pinhel MS, Quinhoneiro DC et al. LEP -2548G>A Polymorphism of the Leptin Gene and Its Influence on the Lipid Profile in Obese Individuals. J Nutrigenet Nutrigenomics. 2014; 7(4-6): 225-31. doi: 10.1159/000371767. |
| [17] | Cao L, Mou S, Fang W et al. Correlational studies on insulin resistance and leptin gene polymorphisms in peritoneal dialysis patients. Iran J Basic Med Sci. 2015; 18(9): 878-86. |
| [18] | Klemettilä JP, Kampman O, Seppälä N et al. Association study of the HTR2C, leptin and adiponectin genes and serum marker analyses in clozapine treated long-term patients with schizophrenia. Eur Psychiatry. 2015; 30(2): 296-302. doi: 10.1016/j.eurpsy.2014.08.006. |
| [19] | Shabana, Hasnain S. Leptin promoter variant G2548A is associated with serum leptin and HDL-C levels in a case control observational study in association with obesity in a Pakistani cohort. J Biosci. 2016; 41(2): 251-5. doi: 10.1007/s12038-016-9612-2. |
| [20] | Yang M, Peng S, Li W et al. Relationships between plasma leptin levels, leptin G2548A, leptin receptor Gln223Arg polymorphisms and gestational diabetes mellitus in Chinese population. Sci Rep. 2016; 6: 23948. doi: 10.1038/srep23948. |
| [21] | Chen J, He J, Chen JH et al. The Study on the relationship between the G2548A polymorphism of leptin gene and its association with environmental factors and cholesterol stones. J Sichun Univ(Med Sci edi). 2017; 48(03): 410-7. doi: 10.13464/j.scuxbyxb.2017.03.017. |
| [22] | Martins MC, Trujillo J, Farias DR et al. Polymorphisms in the leptin (rs7799039) gene are associated with an increased risk of excessive gestational weight gain but not with leptin concentration during pregnancy. Nutr Res. 2017; 47: 53-62. doi: 10.1016/j.nutres.2017.09.003. |
| [23] | Li K, Liu Y, Venners SA et al. Effects of LEP G2548A and LEPR Q223R Polymorphisms on Serum Lipids and Response to Simvastatin Treatment in Chinese Patients With Primary Hyperlipidemia. Clin Appl Thromb Hemost. 2017; 23(4): 336-344. doi: 10.1177/1076029616638504. |
| [24] | Zayani N, Omezzine A, Boumaiza I et al. Association of ADIPOQ, leptin, LEPR, and resistin polymorphisms with obesity parameters in Hammam Sousse Sahloul Heart Study. J Clin Lab Anal. 2017; 31(6): e22148. doi: 10.1002/jcla.22148. |
| [25] | Dagdan B, Chuluun-Erdene A, Sengeragchaa O et al. Leptin Gene G2548A Polymorphism among Mongolians with Metabolic Syndrome. Med Sci (Basel). 2018; 7(1): 3. doi: 10.3390/medsci7010003. |
| [26] | Bains V, Kaur H, Badaruddoza B. Association analysis of polymorphisms in LEP (rs7799039 and rs2167270) and LEPR (rs1137101) gene towards the development of type 2 diabetes in North Indian Punjabi population. Gene. 2020; 754: 144846. doi: 10.1016/j.gene.2020.144846. |
| [27] | Liu HY, Xiao Q. Association analysis between -2548 G/A polymorphism in leptin gene and diabetc kidney disease. Chin J Diabetes. 2020; 28(08): 581-6. doi: 10.3969/j.issn.1006-6187.2020.08.004 |
| [28] | Mohamed AA, Ahmed HH, ElSadek SM et al. A study of leptin and its gene 2548 G/A Rs7799039 single-nucleotide polymorphisms in Egyptian children: A single-center experience. Clin Res Hepatol Gastroenterol. 2021; 45(5): 101724. doi: 10.1016/j.clinre.2021.101724. |
| [29] | Mohamed AA, Hassnine A, Elsayed A et al. Isotretinoin Induced Hyperlipidemia and Impact of Leptin Gene *rs* 7799039 Polymorphism in Safety of Acne Patients. Pharmgenomics Pers Med. 2021; 14: 1679-1687. doi: 10.2147/PGPM.S341723. |
| [30] | Primo D, Izaola O, de Luis D. Leptin gene polymorphism (rs 7799039;G2548A) is associated with changes in lipid profile during a partial meal-replacement hypocaloric diet. J Hum Nutr Diet. 2021; 34(2): 456-463. doi: 10.1111/jhn.12809. |
| [31] | Ali LA, Jemon K, Latif NA et al. LEP G2548A polymorphism is associated with increased serum leptin and insulin resistance among T2DM Malaysian patients. Biomedicine (Taipei). 2022; 12(3): 1-11. doi: 10.37796/2211-8039.1326. |
| [32] | Mohanraj J, D'Souza UJA, Fong SY et al. Association between Leptin (G2548A) and Leptin Receptor (Q223R) Polymorphisms with Plasma Leptin, BMI, Stress, Sleep and Eating Patterns among the Multiethnic Young Malaysian Adult Population from a Healthcare University. Int J Environ Res Public Health. 2022; 19(14): 8862. doi: 10.3390/ijerph19148862. |
| [33] | Sabi EM, Bin Dahman LS, Mohammed AK et al. -2548G>A LEP Polymorphism Is Positively Associated with Increased Leptin and Glucose Levels in Obese Saudi Patients Irrespective of Blood Pressure Status. Medicina (Kaunas). 2022; 58(3): 346. doi: 10.3390/medicina58030346. |
| **NO** | Studies included for *LEPR* rs1137100 variant |
| [1] | Rosmond R, Chagnon YC, Holm G et al. Hypertension in obesity and the leptin receptor gene locus. J Clin Endocrinol Metab. 2000; 85(9): 3126-31. doi: 10.1210/jcem.85.9.6781. |
| [2] | Wauters M, Mertens I, Rankinen T et al. Leptin receptor gene polymorphisms are associated with insulin in obese women with impaired glucose tolerance. J Clin Endocrinol Metab. 2001; 86(7): 3227-32. doi: 10.1210/jcem.86.7.7682. |
| [3] | Wauters M, Mertens I, Chagnon M et al. Polymorphisms in the leptin receptor gene, body composition and fat distribution in overweight and obese women. Int J Obes Relat Metab Disord. 2001; 25(5): 714-20. doi: 10.1038/sj.ijo.0801609. |
| [4] | Salopuro T, Pulkkinen L, Lindström J et al. Genetic variation in leptin receptor gene is associated with type 2 diabetes and body weight: The Finnish Diabetes Prevention Study. Int J Obes (Lond). 2005; 29(10): 1245-51. doi: 10.1038/sj.ijo.0803024. |
| [5] | Chen SH, Li YM, Jiang LL et al. The relationship between leptin receptor Lys109Arg gene polymorphism and non-alcoholic fatty liver disease. Chin J Hepatol. 2006; 14(6): 453-5. doi: 10.3760/j.issn:1007-3418.2006.06.014. |
| [6] | Abete I, Goyenechea E, Crujeiras AB et al. Inflammatory state and stress condition in weight-lowering Lys109Arg LEPR gene polymorphism carriers. Arch Med Res. 2009; 40(4): 306-10. doi: 10.1016/j.arcmed.2009.03.005. |
| [7] | Okada T, Ohzeki T, Nakagawa Y et al. Study Group of Pediatric Obesity and Its related Metabolism. Impact of leptin and leptin-receptor gene polymorphisms on serum lipids in Japanese obese children. Acta Paediatr. 2010; 99(8): 1213-7. doi: 10.1111/j.1651-2227.2010.01778.x. |
| [8] | Saukko M, Kesäniemi YA, Ukkola O. Leptin receptor Lys109Arg and Gln223Arg polymorphisms are associated with early atherosclerosis. Metab Syndr Relat Disord. 2010; 8(5): 425-30. doi: 10.1089/met.2010.0004. |
| [9] | Labayen I, Ruiz JR, Moreno LA et al. The effect of ponderal index at birth on the relationships between common LEP and LEPR polymorphisms and adiposity in adolescents. Obesity (Silver Spring). 2011; 19(10): 2038-45. doi: 10.1038/oby.2011.74. |
| [10] | Aijälä M, Santaniemi M, Bloigu R et al. Leptin receptor Arg109 homozygotes display decreased total mortality as well as lower incidence of cardiovascular disease and related death. Gene. 2014; 534(1): 88-92. doi: 10.1016/j.gene.2013.10.003. |
| [11] | Wu J, Zhuo Q, Chen X et al. Association of leptin receptor gene polymorphrism with metabolic syndrome in older Han adults from major cities in China. Journal of Hygiene Research. 2016; 45(3): 376-82. doi:10.19813/j.cnki.weishengyanjiu.2016. 03.034. |
| [12] | Wu J, Zhuo Q, Tian Y et al. Study on the relationship of hypertension in older Han adults with leptin receptor gene rs1137100 and rs1137101 polymorphism. Journal of Capital Medical University. 2017; 46(3): 384-388. doi:10.3969/ j.issn.1006-7795.2017.03.021. |
| **NO** | Studies included for *LEPR* rs1137101 variant |
| [1] | Silver K, Walston J, Chung WK et al. The Gln223Arg and Lys656Asn polymorphisms in the human leptin receptor do not associate with traits related to obesity. Diabetes. 1997; 46(11): 1898-900. doi: 10.2337/diab.46.11.1898. |
| [2] | Chagnon YC, Wilmore JH, Borecki IB et al. Associations between the leptin receptor gene and adiposity in middle-aged Caucasian males from the HERITAGE family study. J Clin Endocrinol Metab. 2000; 85(1): 29-34. doi: 10.1210/jcem.85.1.6263. |
| [3] | Rosmond R, Chagnon YC, Holm G et al. Hypertension in obesity and the leptin receptor gene locus. J Clin Endocrinol Metab. 2000; 85(9): 3126-31. doi: 10.1210/jcem.85.9.6781. |
| [4] | Ukkola O, Tremblay A, Després JP et al. Leptin receptor Gln223Arg variant is associated with a cluster of metabolic abnormalities in response to long-term overfeeding. J Intern Med. 2000;248(5):435-9. doi: 10.1046/j.1365-2796.2000.00751.x. |
| [5] | Quinton ND, Lee AJ, Ross RJ et al. A single nucleotide polymorphism (SNP) in the leptin receptor is associated with BMI, fat mass and leptin levels in postmenopausal Caucasian women. Hum Genet. 2001; 108(3): 233-6. doi: 10.1007/s004390100468. |
| [6] | Wauters M, Mertens I, Chagnon M et al. Polymorphisms in the leptin receptor gene, body composition and fat distribution in overweight and obese women. Int J Obes Relat Metab Disord. 2001; 25(5): 714-20. doi: 10.1038/sj.ijo.0801609. |
| [7] | Wauters M, Mertens I, Rankinen T et al.Leptin receptor gene polymorphisms are associated with insulin in obese women with impaired glucose tolerance. J Clin Endocrinol Metab. 2001; 86(7): 3227-32. doi: 10.1210/jcem.86.7.7682. |
| [8] | Yiannakouris N, Yannakoulia M, Melistas L et al. The Q223R polymorphism of the leptin receptor gene is significantly associated with obesity and predicts a small percentage of body weight and body composition variability. J Clin Endocrinol Metab. 2001; 86(9): 4434-9. doi: 10.1210/jcem.86.9.7842. |
| [9] | Stefan N, Vozarova B, Del Parigi A et al. The Gln223Arg polymorphism of the leptin receptor in Pima Indians: influence on energy expenditure, physical activity and lipid metabolism. Int J Obes Relat Metab Disord. 2002; 26(12): 1629-32. doi: 10.1038/sj.ijo.0802161. |
| [10] | Chiu KC, Chu A, Chuang LM et al. Association of leptin receptor polymorphism with insulin resistance. Eur J Endocrinol. 2004; 150(5): 725-9. doi: 10.1530/eje.0.1500725. |
| [11] | Guízar-Mendoza JM, Amador-Licona N, Flores-Martínez SE et al. Association analysis of the Gln223Arg polymorphism in the human leptin receptor gene, and traits related to obesity in Mexican adolescents. J Hum Hypertens. 2005; 19(5): 341-6. doi: 10.1038/sj.jhh.1001824. |
| [12] | Méndez-Sánchez N, Bermejo-Martínez L, Chávez-Tapia NC et al. Obesity-related leptin receptor polymorphisms and gallstones disease. Ann Hepatol. 2006; 5(2): 97-102. |
| [13] | Van der Vleuten GM, Kluijtmans LA, Hijmans A et al. The Gln223Arg polymorphism in the leptin receptor is associated with familial combined hyperlipidemia. Int J Obes (Lond). 2006; 30(6): 892-8. doi: 10.1038/sj.ijo.0803234. |
| [14] | Zhao LS, Xiang GD, Tang Y et al. Association of Gln 223 Arg Variant in Leptin Receptor Gene with Hypertension Complicated with Obesity in Wuhan Population. Chin J Prev Contr Chron Non-commun Dis. 2007; 15(2): 91-4. doi: 10.3969/j.issn.1004-6194.2007. 02.004. |
| [15] | Masuo K, Straznicky NE, Lambert GW et al. Leptin-receptor polymorphisms relate to obesity through blunted leptin-mediated sympathetic nerve activation in a Caucasian male population. Hypertens Res. 2008; 31(6): 1093-100. doi: 10.1291/hypres.31.1093. |
| [16] | Zhao LS, Xiang GD, Tang Y et al. Associationof Gln223 Arg Variant in Leptin Receptor Gene in Type2 Diabetes in Wuhan“Han”Population. Military Medical Journal of South China. 2008; 22(2): 25-9. doi: 0.13730/j.1009-2595.2008.02.013. |
| [17] | Ben Ali S, Kallel A, Sediri Y et al. LEPR p.Q223R Polymorphism influences plasma leptin levels and body mass index in Tunisian obese patients. Arch Med Res. 2009; 40(3): 186-90. doi: 10.1016/j.arcmed.2009.02.008. |
| [18] | Constantin A, Costache G, Sima AV et al. Leptin G-2548A and leptin receptor Q223R gene polymorphisms are not associated with obesity in Romanian subjects. Biochem Biophys Res Commun. 2010; 391(1): 282-6. doi: 10.1016/j.bbrc.2009.11.050. |
| [19] | Riestra P, García-Anguita A, Schoppen S et al. Sex-specific association between leptin receptor polymorphisms and leptin levels and BMI in healthy adolescents. Acta Paediatr. 2010; 99(10): 1527-30. doi: 10.1111/j.1651-2227.2010.01877.x. |
| [20] | Saukko M, Kesäniemi YA, Ukkola O. Leptin receptor Lys109Arg and Gln223Arg polymorphisms are associated with early atherosclerosis. Metab Syndr Relat Disord. 2010; 8(5): 425-30. doi: 10.1089/met.2010.0004. |
| [21] | Labayen I, Ruiz JR, Moreno LA et al. The effect of ponderal index at birth on the relationships between common LEP and LEPR polymorphisms and adiposity in adolescents. Obesity (Silver Spring). 2011; 19(10): 2038-45. doi: 10.1038/oby.2011.74. |
| [22] | Sun H, Miao CQ , Zhao XW et al. LEPR Gene Gln223Arg Polymorphism in Chinese Families with Type 2 Diabetes. Progress in Modern Biomedicine. 2011; 11(24): 4852-6. doi: 10.13241/j.cnki.pmb.2011.24.028. |
| [23] | Angel-Chávez LI, Tene-Pérez CE, Castro E. Leptin receptor gene K656N polymorphism is associated with low body fat levels and elevated high-density cholesterol levels in Mexican children and adolescents. Endocr Res. 2012; 37(3): 124-34. doi: 10.3109/07435800. 2011.648360. |
| [24] | Boumaiza I, Omezzine A, Rejeb J et al. Relationship between leptin G2548A and leptin receptor Q223R gene polymorphisms and obesity and metabolic syndrome risk in Tunisian volunteers. Genet Test Mol Biomarkers. 2012; 16(7): 726-33. doi: 10.1089/gtmb.2011. 0324. |
| [25] | Huuskonen A, Lappalainen J, Oksala N et al. Aerobic fitness does not modify the effect of FTO variation on body composition traits. PLoS One. 2012; 7(12): e51635. doi: 10.1371/journal.pone.0051635. |
| [26] | Jackson KG, Delgado-Lista J, Gill R et al. The leptin receptor Gln223Arg polymorphism (rs1137101) mediates the postprandial lipaemic response, but only in males. Atherosclerosis. 2012; 225(1): 135-41. doi: 10.1016/j.atherosclerosis.2012.08.035. |
| [27] | Becer E, Mehmetçik G, Bareke H et al. Association of leptin receptor gene Q223R polymorphism on lipid profiles in comparison study between obese and non-obese subjects. Gene. 2013; 529(1): 16-20. doi: 10.1016/j.gene.2013.08.003. |
| [28] | Zandoná MR, Rodrigues RO, Albiero G et al. Polymorphisms in LEPR, PPARG and APM1 genes: associations with energy intake and metabolic traits in young children. Arq Bras Endocrinol Metabol. 2013; 57(8): 603-11. doi: 10.1590/s0004-27302013000800004. |
| [29] | Zheng H, Xie N, Xu H et al. Association of Gln223Arg polymorphism of the leptin receptor with hypertensive left ventricular hypertrophy. Folia Biol (Praha). 2013; 59(6): 246-52. |
| [30] | Aijälä M, Santaniemi M, Bloigu R et al. Leptin receptor Arg109 homozygotes display decreased total mortality as well as lower incidence of cardiovascular disease and related death. Gene. 2014; 534(1): 88-92. doi: 10.1016/j.gene.2013.10.003. |
| [31] | Al-Azzam SI, Khabour OF, Alzoubi KH et al. The effect of leptin promoter and leptin receptor gene polymorphisms on lipid profile among the diabetic population: modulations by atorvastatin treatment and environmental factors. J Endocrinol Invest. 2014; 37(9): 835-42. doi: 10.1007/s40618-014-0113-6. |
| [32] | Fan SH, Say YH. Leptin and leptin receptor gene polymorphisms and their association with plasma leptin levels and obesity in a multi-ethnic Malaysian suburban population. J Physiol Anthropol. 2014; 33(1): 15. doi: 10.1186/1880-6805-33-15. |
| [33] | Murakami H, Iemitsu M, Fuku N et al. The Q223R polymorphism in the leptin receptor associates with objectively measured light physical activity in free-living Japanese. Physiol Behav. 2014; 129: 199-204. doi: 10.1016/j.physbeh.2014.02.053. |
| [34] | Chen HL, Wang Q, Li JF et al. Influence of leptin recptor gene Gln223Arg variation on ambulatory blood pressure in patients with metabolism syndrome. Journal of ChongQing Medicine. 2016; 45(9): 1201-5. doi: 10.3969/j.issn.1671-8348.2016.09.016. |
| [35] | Wu J, Zhuo Q, Chen X et al.Association of leptin receptor gene polymorphrism with metabolic syndrome in older Han adults from major cities in China. Journal of Hygiene Research. 2016; 45(3): 376-82. doi:10.19813/j.cnki.weishengyanjiu.2016.03.034. |
| [36] | Martins MC, Trujillo J, Farias DR et al. Polymorphisms in the leptin (rs7799039) gene are associated with an increased risk of excessive gestational weight gain but not with leptin concentration during pregnancy. Nutr Res. 2017; 47: 53-62. doi: 10.1016/j.nutres.2017.09.003. |
| [37] | Wu JH, Zhuo Q, Tian Y et al. Study on the relationship of hypertension in older Han adults with leptin receptor gene rs1137100 and rs1137101 polymorphism. Journal of Capital Medical University. 2017; 38(03): 439-45. doi: 10.3969/j.issn.1006-7795.2017.03.021. |
| [38] | Zayani N, Omezzine A, Boumaiza I et al. Association of ADIPOQ, leptin, LEPR, and resistin polymorphisms with obesity parameters in Hammam Sousse Sahloul Heart Study. J Clin Lab Anal. 2017; 31(6): e22148. doi: 10.1002/jcla.22148. |
| [39] | Daghestani M, Purohit R, Daghestani M et al. Molecular dynamic (MD) studies on Gln233Arg (rs1137101) polymorphism of leptin receptor gene and associated variations in the anthropometric and metabolic profiles of Saudi women. PLoS One. 2019; 14(2): e0211381. doi: 10.1371/journal.pone.0211381. |
| [40] | Daghestani MH, Daghestani MH, Daghistani MH et al. The influence of the rs1137101 genotypes of leptin receptor gene on the demographic and metabolic profile of normal Saudi females and those suffering from polycystic ovarian syndrome. BMC Womens Health. 2019; 19(1): 10. doi: 10.1186/s12905-018-0706-x. |
| [41] | Zheng WW, Zahng H, Zhou XH et al. Association of RETN, LEPR and ADIPOQ polymorphisms with the risk of type 2 diabetes and lipid metabolism in Chinese Han population. Journal of WenZhou Medical University. 2019; 49(11): 807-13. doi: 10.3969/j.issn. 2095-9400.2019.11.005. |
| [42] | Bains V, Kaur H, Badaruddoza B. Association analysis of polymorphisms in LEP (rs7799039 and rs2167270) and LEPR (rs1137101) gene towards the development of type 2 diabetes in North Indian Punjabi population. Gene. 2020; 754: 144846. doi: 10.1016/j.gene.2020.144846. |
| [43] | Diéguez-Campa CE, Angel-Chávez LI, Reyes-Ruvalcaba D et al. Leptin Levels and Q223R Leptin Receptor Gene Polymorphism in Obese Mexican Young Adults. EJIFCC. 2020; 31(3): 197-207. |
| [44] | Illangasekera YA, Kumarasiri PVR, Fernando DJ et al. Association of the leptin receptor Q223R (rs1137101) polymorphism with obesity measures in Sri Lankans. BMC Res Notes. 2020; 13(1): 34. doi: 10.1186/s13104-020-4898-4. |
| [45] | Ali EMM, Diab T, Elsaid A et al. Fat mass and obesity-associated (FTO) and leptin receptor (LEPR) gene polymorphisms in Egyptian obese subjects. Arch Physiol Biochem. 2021; 127(1): 28-36. doi: 10.1080/13813455.2019.1573841. |
| [46] | Hu YY, Li LP, Zhang CL et al. The relationship between Gln223Arg polymorphism ofthe leptin receptor gene and patients with Type 2Diabetes the relationship between mellitus complicated with Hyperuricemia and the evaluation of the therapeutic effect of metformin. Labeled Immunoassays and Clinical Medicine. 2021; 28(7): 1155-60. doi: 10.11748/bjmy.issn.1006-1703.2021.07.016. |
| [47] | Mohanraj J, D'Souza UJA, Fong SY et al. Association between Leptin (G2548A) and Leptin Receptor (Q223R) Polymorphisms with Plasma Leptin, BMI, Stress, Sleep and Eating Patterns among the Multiethnic Young Malaysian Adult Population from a Healthcare University. Int J Environ Res Public Health. 2022; 19(14): 8862. doi: 10.3390/ijerph19148862. |
| [48] | Sanchez-Murguia T, Torres-Castillo N, Magaña-de la Vega L et al. Role of Leu72Met of GHRL and Gln223Arg of LEPR Variants on Food Intake, Subjective Appetite, and Hunger-Satiety Hormones. Nutrients. 2022; 14(10): 2100. doi: 10.3390/nu14102100. |
| **NO** | Studies included for *LEPR* rs1805094 variant |
| [1] | Rosmond R, Chagnon YC, Holm G et al. Hypertension in obesity and the leptin receptor gene locus. J Clin Endocrinol Metab. 2000; 85(9): 3126-31. doi: 10.1210/jcem.85.9.6781. |
| [2] | Wauters M, Mertens I, Chagnon M et al. Polymorphisms in the leptin receptor gene, body composition and fat distribution in overweight and obese women. Int J Obes Relat Metab Disord. 2001; 25(5): 714-20. doi: 10.1038/sj.ijo.0801609. |
| [3] | Wauters M, Mertens I, Rankinen T et al. Leptin receptor gene polymorphisms are associated with insulin in obese women with impaired glucose tolerance. J Clin Endocrinol Metab. 2001; 86(7): 3227-32. doi: 10.1210/jcem.86.7.7682. |
| [4] | De Luis Roman D, de la Fuente RA, Sagrado MG et al. Leptin receptor Lys656Asn polymorphism is associated with decreased leptin response and weight loss secondary to a lifestyle modification in obese patients. Arch Med Res. 2006; 37(7): 854-9. doi: 10.1016/j.arcmed.2006.03.009. |
| [5] | De Luis DA, Aller R, Izaola O et al. Influence of Lys656Asn polymorphism of leptin receptor gene on leptin response secondary to two hypocaloric diets: a randomized clinical trial. Ann Nutr Metab. 2008; 52(3): 209-14. doi: 10.1159/000138125. |
| [6] | De Luis DA, Gonzalez Sagrado M, Aller R et al. Influence of Lys656Asn polymorphism of leptin receptor gene on insulin resistance in patients with diabetes mellitus type 2.Diabetes Res Clin Pract. 2008; 81(3): e9-e11. doi: 10.1016/j.diabres.2008.06.002. |
| [7] | De Luis DA, Gonzalez Sagrado M, Aller R et al. Influence of Lys656Asn polymorphism of the leptin receptor gene on insulin resistance in nondiabetic obese patients. J Diabetes Complications. 2008; 22(3): 199-204. doi: 10.1016/j.jdiacomp.2006.10.006. |
| [8] | Masuo K, Straznicky NE, Lambert GW et al. Leptin-receptor polymorphisms relate to obesity through blunted leptin-mediated sympathetic nerve activation in a Caucasian male population. Hypertens Res. 2008; 31(6): 1093-100. doi: 10.1291/hypres.31.1093. |
| [9] | Pérez-Castrillón JL, Vega G, Abad L et al. Atorvastatin and BMD in coronary syndrome role of Lys656Asn polymorphism of leptin receptor gene. Endocr J. 2009; 56(2): 221-5. doi: 10.1507/endocrj.k08e-269. |
| [10] | De Luis DA, Aller R, Sagrado MG et al. Influence of lys656asn polymorphism of leptin receptor gene on surgical results of biliopancreatic diversion. J Gastrointest Surg. 2010; 14(5): 899-903. doi: 10.1007/s11605-010-1181-3. |
| [11] | Murugesan D, Arunachalam T, Ramamurthy V et al. Association of polymorphisms in leptin receptor gene with obesity and type 2 diabetes in the local population of Coimbatore. Indian J Hum Genet. 2010; 16(2): 72-7. doi: 10.4103/0971-6866.69350. |
| [12] | Riestra P, García-Anguita A, Schoppen S et al. Sex-specific association between leptin receptor polymorphisms and leptin levels and BMI in healthy adolescents. Acta Paediatr. 2010; 99(10): 1527-30. doi: 10.1111/j.1651-2227.2010.01877.x. |
| [13] | Labayen I, Ruiz JR, Moreno LA et al. The effect of ponderal index at birth on the relationships between common LEP and LEPR polymorphisms and adiposity in adolescents. Obesity (Silver Spring). 2011; 19(10): 2038-45. doi: 10.1038/oby.2011.74. |
| [14] | Angel-Chávez LI, Tene-Pérez CE, Castro E. Leptin receptor gene K656N polymorphism is associated with low body fat levels and elevated high-density cholesterol levels in Mexican children and adolescents. Endocr Res. 2012; 37(3): 124-34. doi: 10.3109/07435800.2011.648360. |
| [15] | Huuskonen A, Lappalainen J, Oksala N et al. Aerobic fitness does not modify the effect of FTO variation on body composition traits. PLoS One. 2012; 7(12): e51635. doi: 10.1371/journal.pone.0051635. |
| [16] | De Luis DA, Aller R, Izaola O, Conde R, Eiros Bouza J. Lys656Asn polymorphism of leptin receptor gene is related with leptin changes after a high monounsaturated fat diet in obese patients. J Investig Med. 2013; 61(2): 286-90. doi: 10.2310/JIM.0b013e31827c2e4e. |
| [17] | De Luis DA, Aller R, Izaola O et al. Effect of Lys656Asn Polymorphism of Leptin Receptor Gene on Cardiovascular Risk Factors and Serum Adipokine Levels after a High Polyunsaturated Fat Diet in Obese Patients. J Clin Lab Anal. 2015; 29(6): 432-6. doi: 10.1002/jcla.21790. |
| [18] | Tang H, Zhang Z, Li Z et al. High-Carbohydrate/Low-Fat Diet-Induced Gender-Specific Serum Lipid Profile Changes Are Associated with LEPR Polymorphisms in Chinese Youth. Ann Nutr Metab. 2017; 70(1): 1-8. doi: 10.1159/000455165. |
| [19] | Foucan L, Bassien-Capsa V, Rambhojan C et al. Influence of K656N Polymorphism of the Leptin Receptor Gene on Obesity-Related Traits in Nondiabetic Afro-Caribbean Individuals. Metab Syndr Relat Disord. 2019; 17(4): 197-203. doi: 10.1089/met.2018.0133. |
| [20] | Jang K, Shin G, Yoo HJ et al. Risk Associated with the *LEPR* rs8179183 GG Genotype in a Female Korean Population with Obesity. Antioxidants (Basel). 2020; 9(6): 497. doi: 10.3390/antiox9060497. |

**Table S2.** Characteristics of the studies included in the meta-analysis for *LEP* rs7799039 variant.

| **Authors, reference** | **Publication year** | **Ethnicity** | **Gender** | **Subjects** | **Age**  **(Mean**±SD **or age range**) | **Outcomes** |
| --- | --- | --- | --- | --- | --- | --- |
| Jiang et al. [1] | 2007 | Asian | M/F | Hyperuricemia patients/control subjects | 39.87±9.70 | Leptin/glucose/insulin/HOMA-IR/triglycerides |
| Liu et al. [2] | 2008 | Asian | M/F | T2DM patients | 48.2±10.5 | Insulin/HOMA-IR/triglycerides/HDL-C |
| Lai et al. [3] | 2008 | Asian | M/F | NAFLD patients | 55±13 | HOMA-IR |
| Ben et al. [4] | 2009 | African | M/F | Overweight/obesity patients/control subjects | 48±8.7 | Leptin/glucose/insulin/ HOMA-IR/triglycerides/TC/LDL-C/HDL-C |
| Genelhu et al. [5] | 2009 | Latin American | M/F | Overweight/obesity patients | 45.2±12.3 | Leptin/glucose/insulin/HOMA-IR/triglycerides/TC/LDL-C/HDL-C |
| Constantin et al. [6] | 2010 | Caucasian | M/F | Overweight/obesity patients/control subjects | 46±11 | Leptin/glucose/insulin/HOMA-IR/triglycerides/TC/LDL-C/HDL-C |
| Hinuy et al. [7] | 2010 | Latin American | M/F | Overweight/obesity patients/control subjects | 49±14 | Leptin/glucose/triglycerides/TC/LDL-C/HDL-C |
| Riestra et al. [8] | 2010 | Caucasian | M/F | General subjects | 14±2 | Leptin |
| Abdel et al. [9] | 2011 | African | M/F | Psoriasis patients | Unknown | Glucose/triglycerides/TC/LDL-C/HDL-C |
| Boumaiza et al. [10] | 2012 | African | M/F | Overweight/obesity patients/control subjects | 45.76±12.35 | Glucose/insulin/HOMA-IR/triglycerides/TC/HDL-C |
| Han et al. [11] | 2012 | Asian | M | Obstructive sleep apnea-hypopnea syndrome patients | 46±10 | Leptin |
| Huuskonen et al. [12] | 2012 | Caucasian | M | General subjects | 25±5 | Leptin |
| Tavil et al. [13] | 2012 | Asian | M/F | Acute leukemia patients/control subjects | 6.76±4.90 | Leptin/insulin |
| AI-Azzam et al. [14] | 2014 | Asian | M/F | T2DM patients | 56.01±9.67 | Triglycerides/TC/LDL-C/HDL-C |
| Fan et al. [15] | 2014 | Asian | M/F | Overweight/obesity patients/control subjects | 52.4±13.7 | Leptin/triglycerides |
| Ferreira-Julio et al. [16] | 2014 | Latin American | M/F | Overweight/obesity patients/control subjects | 46.97±13.24 | Glucose/triglycerides/TC/LDL-C/HDL-C/ |
| Cao et al. [17] | 2015 | Asian | M/F | Peritoneal dialysis patients | 55±15 | Leptin/HOMA-IR/triglycerides/TC/LDL-C/HDL-C |
| Klemettila et al. [18] | 2015 | Caucasian | M/F | Schizophrenia patients | 20-67 | Leptin |
| Shabana et al. [19] | 2016 | Asian | M/F | Overweight/obesity patients/control subjects | 10-78 | Glucose/triglycerides/TC/LDL-C/HDL-C |
| Yang et al. [20] | 2016 | Asian | F | T2DM patients/control subjects | 29.08±4.36 | Leptin/insulin/HOMA-IR |
| Chen et al. [21] | 2017 | Asian | M/F | Cholesterol gallstone patients/control subjects | 43.59±12.72 | Leptin |
| Martins et al. [22] | 2017 | Latin American | M/F | General subjects | 20-40 | Leptin |
| Li et al. [23] | 2017 | Asian | M/F | Primary hyperlipidemia patients | 52.16±8.58 | Triglycerides/TC/LDL-C/HDL-C |
| Zayani et al. [24] | 2017 | African | M/F | General subjects | 20-70 | Glucose/TC/LDL-C/HDL-C |
| Dagdan et al. [25] | 2018 | Asian | M/F | MS patients | 41.7±11.3 | TC/LDL-C/HDL-C |
| Bains et al. [26] | 2020 | Asian | M/F | General subjects | 45.73±8.34 | Glucose/triglycerides/TC/LDL-C/HDL-C |
| Liu et al. [27] | 2020 | Asian | Male | T2DM/diabetes kidney disease patients | 56.95±5.88 | Glucose/insulin/triglycerides/TC/LDL-C/HDL-C |
| Mohamed et al. [28] | 2021 | African | M/F | Overweight/obesity patients/control subjects | 8.68±1.07 | Glucose/insulin/HOMA-IR/triglycerides/TC/LDL-C/HDL-C |
| Mohamed et al. [29] | 2021 | African | M/F | Acne patients | 24.93±5.96 | Triglycerides/TC/LDL-C/HDL-C |
| Primo et al. [30] | 2021 | Caucasian | M/F | Overweight/obesity patients | 40-65 | Glucose/insulin/HOMA-IR/triglycerides/TC/LDL-C/HDL-C |
| Ali et al. [31] | 2022 | Asian | M/F | T2DM patients/control subjects | 44.75±13.2 | Glucose/insulin/HOMA-IR |
| Mohanralj et al. [32] | 2022 | Asian | M/F | General subjects | 18-24 | Leptin |
| Sabi et al. [33] | 2022 | Asian | M/F | Overweight/obesity patients/hypertensives /T2DM patients/control subjects | 48.26±5.86 | Leptin/glucose/insulin/HOMA-IR/triglycerides/TC/LDL-C/HDL-C |

LEP, leptin; SD, standard deviation; M, male; F, female; HOMA-IR, homeostasis model assessment of insulin resistance; TC, total cholesterol; LDL-C, low-density lipoprotein cholesterol; HDL-C, high-density lipoprotein cholesterol; T2DM, type 2 diabetes mellitus; MS, metabolic syndrome; NAFLD, non-alcoholic fatty liver disease.

**Table S3.** Original data for leptin and glucose metabolism markers by the genotypes of *LEP* rs7799039 variant.

| **Authors, reference** | **Subjects** | **N** | | **Leptin, ng/ml** | | **Glucose, mg/dL** | | **Insulin, μU/mL** | | **HOMA-IR** | |
| --- | --- | --- | --- | --- | --- | --- | --- | --- | --- | --- | --- |
|  |  | GG | GA+AA | GG | GA+AA | GG | GA+AA | GG | GA+AA | GG | GA+AA |
| Jiang et al. [1] | Hyperuricemia/control subjects | 40 | 281 | 3.01±3.31 | 4.21±3.53 | 102.42±24.84 | 103.69±24.31 | 12.91±7.67 | 15.45±11.04 | 3.42±3.02 | 3.95±2.98 |
| Liu et al. [2] | T2DM patients | 14 | 231 | - | - | 156.24±68.22 | 150.67±50.97 | 9.03±5.33 | 10.18±12.31 | 3.20±2.09 | 2.96±4.21 |
| Lai et al. [3] | NAFLD patients | 7 | 100 | - | - | - | - | - | - | 1.4±0.5 | 2.0±0.6 |
| Ben et al. [4] | Overweight/obesity patients | 97 | 132 | 21.37±11.72 | 16.96±8.27 | 117.1±40.0 | 127.8±59.7 | 12.75±9.07 | 10.89±7.23 | 3.76±3.05 | 3.32±2.60 |
|  | Control subjects | 113 | 138 | 7.84±4.97 | 4.67±4.09 | 96.97±35.97 | 92.73±25.72 | 5.31±5.21 | 5.00±4.74 | 1.24±1.20 | 1.14±1.12 |
| Genelhu et al. [5] | Overweight/obesity patients | 78 | 62 | 42.1±25.2 | 42.26±32.77 | 103.7±21.1 | 108.38±31.51 | 24.2±12.2 | 22.76±11.61 | 6.2 ± 3.4 | 6.24±4.26 |
| Constantin et al. [6] | Overweight/obesity/control subjects | 81 | 121 | 17.2±6.6 | 13.2±4.9 | 143.7±65.4 | 145±67.8 | 8.6±3.8 | 8.60±4.0 | 3.2±2.6 | 3.29±2.63 |
| Hinuy et al. [7] | Overweight/obesity/control subjects | 75 | 135 | 26.1±20.6 | 21.21±5.25 | 104±27 | 102.21±64.20 | - | - | - | - |
| Riestra et al. [8] | General subjects | 120 | 221 | 7.2±9.5 | 5.46±7.18 | - | - | - | - | - | - |
|  | General subjects | 123 | 265 | 17.4±10.8 | 15.34±9.89 | - | - | - | - | - | - |
| Abdel et al.[9] | Psoriasis patients | 36 | 58 | - | - | 120.06±15.3 | 106.37±20.32 | - | - | - | - |
| Boumaiza et al. [10] | Overweight/obesity patients/control subjects | 130 | 199 | - | - | 122.04±65.16 | 113.58±43.92 | 6.77±5.16 | 8.11±6.32 | 1.85±1.60 | 2.1±2.07 |
| Han et al. [11] | Obstructive sleep apnea-hypopnea syndrome patients | 36 | 249 | 18.87±3.95 | 18.46±3.17 | - | - | - | - | - | - |
| Huuskonen et al. [12] | Control subjects | 176 | 537 | 3.73±3.68 | 3.76±3.87 | - | - | - | - | - | - |
| Tavil et al. [13] | Control subjects | 14 | 56 | 8.6±10.9 | 5.33±5.21 | - | - | 9.1±8.3 | 7.04±9.95 | - | - |
|  | Acute leukemia patients | 15 | 57 | 5.1±3 | 4.89±3.53 | - | - | 3.7±2.0 | 5.4±5.08 | - | - |
| Fan et al. [15] | Overweight/obesity/control subjects | 48 | 360 | 32.40±37.75 | 27.22±35.63 | - | - | - | - | - | - |
| Ferreira-Julio et al. [16] | Overweight/obesity patients | 27 | 109 | - | - | 117.7±57.6 | 101±32.1 | - | - | - | - |
|  | Control subjects | 25 | 51 | - | - | 98.1±32.8 | 96.2±31.1 | - | - | - | - |
| Cao et al. [17] | Peritoneal dialysis patients | 2 | 77 | 41.13±3.79 | 16.71±10.4 | - | - | - | - | 6.51±4.31 | 4.29±7.47 |
| Klemettila et al. [18] | Schizophrenia patients | 40 | 139 | 28.41±32.38 | 27.87±32.70 | - | - | - | - | - | - |
| Shabana et al. [19] | Overweight/obesity/control subjects | 202 | 273 | - | - | 105.11±186.47 | 97.78±134.97 | - | - | - | - |
| Yang et al. [20] | T2DM patients/control subjects | 47 | 648 | 24.95±45.74 | 26.52±98.19 | - | - | 7.68±4.17 | 7.27±16.63 | 1.47±1.19 | 1.44±3.88 |
| Chen et al. [21] | Cholesterol gallstone patients/control subjects | 52 | 548 | 7.91±1.93 | 8.10±2.06 | - | - | - | - | - | - |
| Martins et al. [22] | General subjects | 68 | 76 | 15.2±9.71 | 15.73±11.38 | - | - | - | - | - | - |
| Zayani et al. [24] | General subjects | 234 | 887 | - | - | 102.06±34.92 | 102.36±32.82 | - | - | - | - |
| Bains et al. [26] | General subjects | 280 | 120 | - | - | 85.22±12.57 | 86.69±12.23 | - | - | - | - |
| Liu et al. [27] | T2DM patients | 60 | 140 | - | - | 150.66±14.22 | 148.72±17.98 | 10.22±4.96 | 10.61±4.53 | - | - |
|  | Diabetes kidney disease patients | 30 | 170 | - | - | 145.98±12.78 | 148.44±19.37 | 11.09±2.33 | 12.99±4.74 | - | - |
| Mohamed et al. [28] | Control subjects | 50 | 36 | - | - | 91±6.6 | 92.58±5.05 | 18.8±6.8 | 23.28±6.65 | 4.2±1.6 | 5.25±1.53 |
|  | Overweight/obesity patients | 78 | 65 | - | - | 101.3±15 | 100.66±16.09 | 23.2±6.8 | 24.76±6.68 | 5.8±2 | 6.09±1.84 |
| Primo et al. [30] | Overweight/obesity patients | 26 | 96 | - | - | 102.3±6.1 | 100.1±7.1 | 15.7±1.9 | 16.1±1.1 | 4.1±0.3 | 4.2±0.2 |
| Ali et al. [31] | Control subjects | 80 | 70 | - | - | 87.66±9 | 86.04±7.74 | 11.14±5.41 | 11.35±5.8 | 2.42±1.25 | 2.43±1.34 |
|  | T2DM patients | 51 | 99 | - | - | 207±52.92 | 193.01±45.94 | 18.22±7.76 | 20.66±9.33 | 9.17±4.55 | 9.82±5.31 |
| Mohanralj et al. [32] | General subjects | 10 | 53 | 3.18±0.79 | 2.92±0.67 | - | - | - | - | - | - |
| Sabi et al. [33] | Control subjects | 23 | 27 | - | - | 104.4±2.88 | 101.16±17.64 | 8.2±1.8 | 10.39±2.33 | 2.2±0.88 | 2.61±1.08 |
|  | Overweight/obesity patients | 32 | 48 | 38.8±2.7 | 39.45±11.46 | 100.8±5.22 | 122.4±7.92 | 11.0±1.1 | 11.45±1.61 | 2.7±0.54 | 3.38±0.74 |
|  | Overweight/obesity/hypertensives/T2DM patients | 25 | 51 | 38.3±2.4 | 38.83±4.91 | 109.8±6.66 | 132.84±10.44 | 11.1±1.0 | 12.80±1.06 | 3.0±0.62 | 4.21±0.74 |

LEP, leptin; HOMA-IR, homeostasis model assessment of insulin resistance; T2DM, type 2 diabetes mellitus; MS, metabolic syndrome; NAFLD, non-alcoholic fatty liver disease.

**Table S4.** Original data for lipid metabolism markers by the genotypes of *LEP* rs7799039 variant.

| **Authors, reference** | **Subjects** | **N** | | **Triglycerides, mg/dL** | | **TC, mg/dL** | | **LDL-C, mg/dL** | | **HDL-C, mg/dL** | |
| --- | --- | --- | --- | --- | --- | --- | --- | --- | --- | --- | --- |
|  |  | GG | GA+AA | GG | GA+AA | GG | GA+AA | GG | GA+AA | GG | GA+AA |
| Jiang et al. [1] | Hyperuricemia patients/control subjects | 40 | 281 | 179.75±159.38 | 197.46±149.64 | - | - | - | - | - | - |
| Liu et al. [2] | T2DM patients | 14 | 231 | 239.07±191.26 | 272.03±331.68 | - | - | 117.56±46.02 | 115.65±40.68 | 71.15±77.73 | 52.4±32.95 |
| Ben et al. [4] | Overweight/obesity patients | 97 | 132 | 133.70±55.78 | 143.44±65.52 | 187.94±29.78 | 193.35±36.74 | 114.85±25.91 | 116.01±31.71 | 44.86±9.67 | 46.02±11.99 |
|  | Control subjects | 113 | 138 | 100.06±45.16 | 97.40±33.65 | 175.18±36.74 | 182.14±34.03 | 104.80±32.87 | 107.89±29.78 | 51.04±12.76 | 53.75±14.69 |
| Genelhu et al. [5] | Overweight/obesity patients | 78 | 62 | 153.1±83.8 | 154.73±83.01 | 201.2±39.5 | 203.12±46.18 | 126±31.6 | 125.51±40.22 | 44.5±9.7 | 46.62±10.87 |
| Constantin et al. [6] | Overweight/obesity/control subjects | 81 | 121 | 135.1±84.9 | 131.4±83.2 | 213.9±50.0 | 213.8±51 | 137.0±41.3 | 131.2±38.2 | 45.1±14.1 | 52.6±15.4 |
| Hinuy et al. [7] | Overweight/obesity/control subjects | 75 | 135 | 153±60 | 137.89±72.67 | 215±42 | 207.93±44.13 | 132±34 | 124.75±36.92 | 53±13 | 55.39±14.57 |
| Abdel et al. [9] | Psoriasis patients | 36 | 58 | 95.78±11.35 | 87.62±10.74 | 294.54±36.03 | 261.7±38.4 | 80.88±6.27 | 78.44±8.97 | 35.07± 4.20 | 38.63±4.23 |
| Boumaiza et al. [10] | Overweight/obesity patients/control subjects | 130 | 199 | 95.63±46.93 | 101.53±50.33 | 172.47±37.12 | 187.22±45.09 | - | - | 47.56±15.08 | 44.31±15.29 |
| AI-Azzam et al. [14] | T2DM patients | 99 | 269 | 234.64±262.98 | 186.68±136.92 | 202.24±51.04 | 191.16±50.88 | 125.29±41.76 | 120.83±42.23 | 47.56±26.30 | 44.81±22.63 |
| Ferreira-Julio et al. [16] | Overweight/obesity patients | 27 | 109 | 138.2±71.4 | 140.1±72.9 | 191.6±44.8 | 187.6±39.4 | 115.7±38.4 | 112.3±29.9 | 44.7±11.0 | 44.5±12.3 |
|  | Control subjects | 25 | 51 | 114.7±46.5 | 106.3±41.1 | 176.9±37.4 | 172±45.2 | 101.7±32.9 | 103.9±34.7 | 52.3±11.7 | 53.3±14.2 |
| Cao et al. [17] | Peritoneal dialysis patients | 2 | 77 | 228.45±112.45 | 196.94±106.44 | 237.43±33.27 | 203.33±46.58 | 148.49±21.27 | 125.6±41.34 | 48.72±16.24 | 45.76±13.72 |
| Shabana et al. [19] | Overweight/obesity/control subjects | 202 | 273 | 206.31±75.26 | 193.78±59.97 | 190.64±38.28 | 199.68±41.67 | 99.00±27.46 | 102.43±27.03 | 57.23±16.63 | 53.21±16.45 |
| Li et al. [23] | Primary hyperlipidemia patients | 7 | 99 | 184.17±78.81 | 189.13±108.23 | 242.07±14.69 | 246.42±20.68 | 123.36±21.27 | 127.49±31.56 | 79.66±27.46 | 72.93±29.22 |
|  | Primary hyperlipidemia patients | 5 | 101 | 156.72±32.76 | 169.01±82.49 | 232.79±42.15 | 261.84±22.51 | 164.35±8.12 | 170.67±18.25 | 49.50±6.96 | 57.18±15.05 |
| Zayani et al. [24] | General subjects | 234 | 887 | - | - | 189.87±42.15 | 189.1±37.07 | 128.77±33.64 | 127.19±30.75 | 44.08±11.60 | 74.14±44.36 |
| Dagdan et al. [25] | MS patients | 98 | 62 | - | - | 156.70±39.08 | 159.25±31.78 | 96.90±36.95 | 93.97±43.18 | 33.89±13.26 | 30.86±10.05 |
| Bains et al. [26] | General subjects | 280 | 120 | 141.94±47.15 | 136.08±36.17 | 172.19±35.03 | 170.36±33.68 | 98.46±32.55 | 98.12±31.25 | 45.34±12.74 | 45.3±13.7 |
| Liu et al. [27] | T2DM patients | 60 | 140 | 610.08±151.41 | 622.22±137.74 | 168.99±64.19 | 173.78±69.26 | 64.58±54.14 | 59.98±45.39 | 78.50±16.63 | 82.17±18.36 |
|  | Diabetes kidney disease patients | 30 | 170 | 313.45±247.93 | 329.00±162.88 | 182.91±61.10 | 183.28±61.99 | 58.78±57.23 | 56.92±50.08 | 78.11±17.79 | 80.67±18.17 |
| Mohamed et al. [28] | Control subjects | 50 | 36 | 144±15.3 | 145.5±22.55 | 147.8±15.4 | 152.62±24.75 | 107.9±11.9 | 105.95±9.36 | 46±9 | 44±6.23 |
|  | Overweight/obesity patients | 78 | 65 | 159±32 | 160.96±32.04 | 172.4±34.3 | 181.07±34.95 | 116±17 | 121.27±22.14 | 41.7±8.8 | 39.9±9.65 |
| Mohamed et al. [29] | Acne patients | 59 | 41 | 149.8±25.4 | 149.27±24.22 | 172.9±23 | 188.44±32.60 | 120.2±16.2 | 118.92±18.51 | 43.7±8.2 | 44.89±8.10 |
| Primo et al. [30] | Overweight/obesity patients | 26 | 96 | 110.4±12.1 | 112.1±13.2 | 217.1±12.7 | 216.9±5.1 | 138.1±4.3 | 134.6±8.1 | 57.4±4.1 | 57.2±5.1 |
| Sabi et al. [33] | Control subjects | 23 | 27 | 141.67±15.05 | 126.62±14.17 | 204.95±46.4 | 208.04±39.06 | 143.08±42.54 | 146.17±31.32 | 29±6.96 | 32.48±6.19 |
|  | Overweight/obesity patients | 32 | 48 | 177.09±88.55 | 169.12±122.19 | 216.55±54.14 | 204.56±38.67 | 150.81±46.4 | 140.37±39.83 | 28.23±6.57 | 26.68±7.73 |
|  | Overweight/obesity/hypertensives/T2DM patients | 25 | 51 | 159.38±17.71 | 176.2±23.02 | 204.95±46.4 | 220.03±41.76 | 139.21±7.73 | 149.27±39.44 | 29.39±6.19 | 33.64±7.35 |

LEP, leptin; TC, total cholesterol; LDL-C, low-density lipoprotein cholesterol; HDL-C, high-density lipoprotein cholesterol; T2DM, type 2 diabetes mellitus; MS, metabolic syndrome.

**Table S5.** Characteristics of the studies included in the meta-analysis for *LEPR* rs1137100 variant.

| **Authors, reference** | **Publication year** | **Ethnicity** | **Gender** | **Subjects** | **Age**  **(Mean**±SD **or age range**) | **Outcomes** |
| --- | --- | --- | --- | --- | --- | --- |
| Rosmond et al. [1] | 2000 | Caucasian | M | General subjects | 51 | Leptin/glucose/insulin/triglycerides/TC/LDL-C/HDL-C |
| Wauters et al. [2] | 2001 | Caucasian | F | Overweight/obesity/T2DM patients/impaired glucose tolerance subjects | 40±9.94 | Glucose/insulin |
| Wauters et al. [3] | 2001 | Caucasian | F | Overweight/obesity patients | 38.69±10.99 | Leptin |
| Salopuro et al. [4] | 2005 | Caucasian | M/F | Impaired glucose tolerance subjects | 55.3±7.1 | Glucose/insulin/HOMA-IR |
| Chen et al. [5] | 2006 | Asian | M/F | NAFLD patients/control subjects | 49.95±13.58 | TC |
| Abete et al. [6] | 2009 | Caucasian | M/F | Overweight/obesity patients | 35±6 | Leptin/glucose/insulin/HOMA-IR/triglycerides/TC/LDL-C/HDL-C/ |
| Okada et al. [7] | 2010 | Asian | M/F | Overweight/obesity patients | 5-17 | Triglycerides/TC/LDL-C/HDL-C |
| Saukko M et al. [8] | 2010 | Caucasian | M/F | General/control subjects | 51.75±6.26 | Glucose/triglycerides/TC/LDL-C/HDL-C |
| Labayen et al. [9] | 2011 | Caucasian | M/F | General/control subjects | 14.8±1.4 | Leptin |
| Aijala et al. [10] | 2014 | Caucasian | M/F | Hypertensive patients/control subjects | 51.29±9.94 | Leptin/LDL-C |
| Wu et al. [11] | 2017 | Asian | M/F | Hypertensive patients/control subjects | 69.3±6.92 | Leptin/glucose/insulin/HOMA-IR/triglycerides/TC/LDL-C/HDL-C |
| Wu et al. [12] | 2016 | Asian | M/F | MS patients/control subjects | 68.23±5.86 | Leptin/glucose/insulin/HOMA-IR/triglycerides/TC/LDL-C/HDL-C |

LEPR, leptin receptor; SD, standard deviation; M, male; F, female; HOMA-IR, homeostasis model assessment of insulin resistance; TC, total cholesterol; LDL-C, low-density lipoprotein cholesterol; HDL-C, high-density lipoprotein cholesterol; T2DM, type 2 diabetes mellitus; MS, metabolic syndrome; NAFLD, non-alcoholic fatty liver disease.

**Table S6.** Original data for leptin and glucose metabolism markers by the genotypes of *LEPR* rs1137100 variant.

| **Authors, reference** | **Subjects** | **N** | | **Leptin, ng/ml** | | **Glucose, mg/dL** | | **Insulin, μU/mL** | | **HOMA-IR** | |
| --- | --- | --- | --- | --- | --- | --- | --- | --- | --- | --- | --- |
|  |  | AA | AG+GG | AA | AG+GG | AA | AG+GG | AA | AG+GG | AA | AG+GG |
| Rosmond et al. [1] | General subjects | 161 | 106 | 6.3±4.4 | 5.96±4.14 | 82.8±18 | 81.98±16.43 | 13.0±10.4 | 12.10±11.67 | - | - |
| Wauters et al. [2] | Overweight/obesity/T2DM patients/impaired glucose tolerance subjects | 29 | 34 | - | - | 97.2±38.7 | 104.4±31.5 | 18.81±10.82 | 21.97±10.88 | - | - |
|  | Overweight/obesity/T2DM patients/impaired glucose tolerance subjects | 7 | 15 | - | - | 100.8±28.62 | 109.8±27.9 | 32.45±12.92 | 17.52±12.23 | - | - |
| Wauters et al. [3] | Overweight/obesity patients | 98 | 100 | 35.8± 13.23 | 36.9±13.5 | - | - | - | - | - | - |
|  | Overweight/obesity patients | 48 | 34 | 50.5±17.71 | 28.6±17.71 | - | - | - | - | - | - |
| Salopuro et al. [4] | Impaired glucose tolerance subjects | 206 | 301 | - | - | 109.8±14.4 | 111.2±13.68 | 12.68 ± 6.19 | 12.75±6.51 | 4.09± 2.23 | 4.11±2.34 |
| Abete et al. [6] | Overweight/obesity patients | 107 | 59 | 22.4±12.4 | 22.1±15.4 | 91.2±7.4 | 91.5±8.2 | 9.7±4.9 | 11.4±9.0 | 2.47±1.53 | 2.35±1.10 |
| Saukko M et al. [8] | General subjects | 213 | 306 | - | - | 79.2±13.32 | 78.81±13.06 | - | - | - | - |
| Labayen et al. [9] | General subjects | 490 | 333 | 21.4±25.1 | 19.12±21.06 | - | - | - | - | - | - |
| Aijala et al. [10] | Hypertensive patients/control subjects | 427 | 601 | 11.4±8.5 | 10.14±7.99 | - | - | - | - | - | - |
| Wu et al. [11] | Hypertensive patients/control subjects | 16 | 506 | 4.65±7.23 | 2.62±4.64 | 84.78±9.9 | 86.61±8.59 | 6.14±2.49 | 5.60±3.73 | 1.30±0.58 | 1.19±0.82 |
| Wu et al. [12] | MS patients  /control subjects | 71 | 2011 | 10.38±11.79 | 11.69±14.43 | 125.82±42.3 | 121.72±47.92 | 6.72±7.96 | 7.39±5.38 | 1.97±2.53 | 2.23±1.67 |

LEPR, leptin receptor; HOMA-IR, homeostasis model assessment of insulin resistance; T2DM, type 2 diabetes mellitus; MS, metabolic syndrome; NAFLD, non-alcoholic fatty liver disease.

**Table S7.** Original data for lipid metabolism markers by the genotypes of *LEP* rs1137100 variant.

| **Authors, reference** | **Subjects** | **N** | | **Triglycerides, mg/dL** | | **TC, mg/dL** | | **LDL-C, mg/dL** | | **HDL-C, mg/dL** | |
| --- | --- | --- | --- | --- | --- | --- | --- | --- | --- | --- | --- |
|  |  | AA | AG+GG | AA | AG+GG | AA | AG+GG | AA | AG+GG | AA | AG+GG |
| Rosmond et al. [1] | General subjects | 161 | 106 | 168.24±97.4 | 146.52±86.58 | 239.75±42.54 | 237.12±41.05 | 158.55±42.54 | 158.91±34.44 | 46.4±11.6 | 51.15±11.66 |
| Chen et al. [5] | NAFLD/control subjects | 3 | 177 | - | - | 197.22±15.47 | 1039.57±315.17 | - | - | - | - |
| Abete et al. [6] | Overweight/obesity patients | 107 | 59 | 105.1±48.2 | 98.4±39.9 | 209.7±38.6 | 196.1±35.0 | 135.4±34.6 | 126.6±33.0 | 53.0±14.2 | 50.2±11.3 |
| Okada et al. [7] | Overweight/obesity patients | 80 | 56 | 109.3±57.24 | 114.21±73.79 | 176.2±34.88 | 193.61±40.71 | 105.1±31.1 | 117.35±33.4 | 49.3±9.84 | 54.88±14.26 |
| Saukko M et al. [8] | General subjects | 213 | 306 | 106.25±32.76 | 108.16±39.61 | 212.69±28.62 | 215.19±44.56 | 127.61±42.92 | 133.15±32.76 | 50.27±14.31 | 54.14±15.22 |
| Aijala et al. [10] | Hypertensive patients/control subjects | 427 | 601 | - | - | - | - | 135.35±38.67 | 136.12±34.8 | - | - |
| Wu et al. [11] | Hypertensive patients/control subjects | 16 | 506 | 100.06±66.41 | 91.79±52.56 | 193.35±46.40 | 168.32±31.68 | 112.14±44.86 | 93.19±26.0 | 59.17±13.53 | 54.98±11.71 |
| Wu et al. [12] | MS patients/control subjects | 71 | 2011 | 178.86±92.97 | 162.77±88.56 | 173.24±40.60 | 178.66±39.91 | 100.16±27.84 | 99.03±33.40 | 40.60±11.21 | 44.62±10.60 |

LEPR, leptin receptor; TC, total cholesterol; LDL-C, low-density lipoprotein cholesterol; HDL-C, high-density lipoprotein cholesterol; T2DM, type 2 diabetes mellitus; MS, metabolic syndrome.

**Table S8.** Characteristics of the studies included in the meta-analysis for *LEPR* rs1137101 variant.

| **Authors, reference** | **Publication year** | **Ethnicity** | **Gender** | **Subjects** | **Age**  **(Mean**±SD **or age range**) | **Outcomes** |
| --- | --- | --- | --- | --- | --- | --- |
| Silver et al. [1] | 1997 | Caucasian | M/F | Overweight/obesity patients | 49.58±15.2 | Glucose/insulin |
| Chagnon et al. [2] | 2000 | Caucasian | M/F | General subjects | 22.15±24.56 | Leptin |
| Rosmond et al. [3] | 2000 | Caucasian | M | General subjects | 51 | Leptin/glucose/insulin/triglycerides/TC/LDL-C/HDL-C |
| Ukkola et al. [4] | 2000 | Caucasian | M | General subjects | 21±2 | Leptin/glucose/insulin/triglycerides/TC/HDL-C |
| Quinton et al. [5] | 2001 | Caucasian | F | Postmenopausal subjects | 67.7±7.42 | Leptin |
| Wauters et al. [6] | 2001 | Caucasian | F | Overweight/obesity patients | 39±10.98 | Leptin |
| Wauters et al. [7] | 2001 | Caucasian | F | Obesity patients | 39±9.94 | Glucose/insulin |
| Yiannakouris et al. [8] | 2001 | Caucasian | M/F | General subjects | 16.74±1.70 | Leptin |
| Stefan et al. [9] | 2002 | Caucasian | M/F | Non-diabetic subjects | 27.32±26.49 | Leptin |
| Chiu et al. [10] | 2004 | Caucasian | M/F | Glucose-tolerant subjects | 26.76±6.11 | Glucose/insulin/triglycerides/TC/LDL-C/HDL-C |
| Guizar-Mendoza et al. [11] | 2005 | Latin American | M/F | Obese patients/lean subjects | 13.99±1.49 | Leptin/insulin |
| Mendez-Sanchez et al. [12] | 2006 | Latin American | M/F | Gallstones patients | 47.59±12.08 | Leptin/glucose/insulin/HOMA-IR/triglycerides/TC/LDL-C/HDL-C |
| Van der Vleuten et al. [13] | 2006 | Caucasian | M/F | General subjects | 40.36±18.05 | Leptin/HOMA-IR/triglycerides/TC/HDL-C |
| Zhao et al. [14] | 2007 | Asian | M/F | Hypertension/overweight/obesity patients | 51.99±5.09 | Triglycerides/TC/LDL-C/HDL-C |
| Masuo et al. [15] | 2008 | Caucasian | M | Obese patients/lean subjects | 46.82±9.27 | Leptin |
| Zhao et al. [16] | 2008 | Asian | M/F | Obesity/T2DM patients | 54.44±4.13 | Triglycerides/TC/LDL-C/HDL-C |
| Ben et al. [17] | 2009 | African | M/F | Obesity patients/control subjects | 46.01±11.4 | Leptin/triglycerides/TC/LDL-C/HDL-C |
| Constantin et al. [18] | 2010 | Caucasian | M/F | Overweight/obesity patients/control subjects | 47±9.98 | Leptin/glucose/insulin/HOMA-IR/triglycerides/TC/LDL-C/HDL-C |
| Riestra et al. [19] | 2010 | Caucasian | M/F | General subjects | 12-16 | Leptin |
| Saukko et al. [20] | 2010 | Caucasian | M/F | General subjects | 51.49±4.13 | Glucose/triglycerides/TC/LDL-C/HDL-C |
| Labayen et al. [21] | 2011 | Caucasian | M/F | General subjects | 14.8±1.4 | Leptin |
| Sun et al. [22] | 2011 | Asian | M/F | T2DM patients/control subjects | 50.37±10.53 | Glucose |
| Angel-Chavez et al. [23] | 2012 | Latin American | M/F | Overweight/obesity patients/control subjects | 11.39±2.99 | Glucose/triglycerides/TC/LDL-C/HDL-C |
| Boumaiza et al. [24] | 2012 | African | M/F | Overweight/obesity patients/control subjects | 48.41±10.92 | Glucose/insulin/HOMA-IR/triglycerides/TC/HDL-C |
| Huuskonen et al. [25] | 2012 | Caucasian | M | General subjects | 25±5 | Leptin |
| Jackson et al. [26] | 2012 | Caucasian | M/F | The postprandial lipaemic response subjects | 54.62±11.62 | Glucose/insulin/HOMA-IR/TC/LDL-C/HDL-C |
| Becer et al. [27] | 2013 | Asian | M/F | Obesity patients/control subjects | 39.65±9.07 | Glucose/HOMA-IR/triglycerides/TC/LDL-C/HDL-C |
| Zandona et al. [28] | 2013 | Latin American | M/F | General subjects | 3-4 | Glucose |
| Zheng et al. [29] | 2013 | Asian | M/F | Hypertensive left ventricular hypertrophy patients | 56.4±8.5 | Leptin/glucose/triglycerides/TC/LDL-C/HDL-C |
| Aijala et al. [30] | 2014 | Caucasian | M/F | Hypertensive patients/control subjects | 51.49±6.02 | LDL-C |
| Al-Azzam et al. [31] | 2014 | Asian | M/F | Diabetic patients | 56.01±9.67 | Triglycerides/TC/LDL-C/HDL-C |
| Fan et al. [32] | 2014 | Asian | M/F | Overweight/obesity patients/control subjects | 52.4±13.7 | Leptin |
| Murakami et al. [33] | 2014 | Asian | M/F | General subjects | 49.09±9.6 | Glucose/triglycerides/HDL-C |
| Chen et al. [34] | 2016 | Asian | M/F | MS patients | 48.16±13.22 | Glucose/insulin/HOMA-IR/triglycerides/TC/LDL-C/HDL-C |
| Wu et al. [35] | 2016 | Asian | M/F | MS patients/control subjects | 74±14 | Leptin/Glucose/insulin/HOMA-IR/triglycerides/TC/LDL-C/HDL-C |
| Martins et al. [36] | 2017 | Latin American | F | Control subjects | 20-40 | Leptin |
| Wu et al. [37] | 2017 | Asian | M/F | Hypertensive patients/control subjects | 78±18 | Leptin/glucose/insulin/HOMA-IR/triglycerides/TC/LDL-C/HDL-C |
| Zayani et al. [38] | 2017 | African | M/F | General subjects | 47.5±16.25 | Glucose/TC/LDL-C/HDL-C |
| Daghestani et al. [39] | 2019 | Asian | F | Obesity patients/control subjects | Unknown | Glucose/insulin/HOMA-IR/triglycerides/TC/LDL-C/HDL-C |
| Daghestani et al. [40] | 2019 | Asian | F | PCOS patients/control subjects | Unknown | Glucose/insulin/HOMA-IR/triglycerides/TC/LDL-C/HDL-C |
| Zheng et al. [41] | 2019 | Asian | M/F | Overweight/obesity patients/control subjects | 49.99±11.48 | Triglycerides/TC/LDL-C/HDL-C |
| Bains et al. [42] | 2020 | Asian | M/F | General subjects | 45.73±8.34 | Glucose/triglycerides/TC/LDL-C/HDL-C |
| Dieguez-Campa et al. [43] | 2020 | Latin American | M/F | Obesity patients/control subjects | 20.98±1.96 | Leptin |
| Illangasekera et al. [44] | 2020 | Asian | M/F | Overweight/obesity patients/control subjects | 47.5±11.2 | Glucose/triglycerides/TC/LDL-C/HDL-C |
| Ali et al. [45] | 2021 | African | M/F | Obesity patients | 41.3±11.8 | Glucose/insulin/HOMA-IR/triglycerides/TC/LDL-C/HDL-C |
| Hu et al. [46] | 2021 | Asian | M/F | T2DM/HUA patients | 57.5±6.2 | Glucose |
| Mohanraj et al. [47] | 2022 | Asian | M/F | General subjects | 18-24 | Leptin |
| Sanchez-Murguia et al. [48] | 2022 | Latin American | M/F | General subjects | 18-25 | Leptin/insulin |

LEPR, leptin receptor; SD, standard deviation; M, male; F, female; HOMA-IR, homeostasis model assessment of insulin resistance; TC, total cholesterol; LDL-C, low-density lipoprotein cholesterol; HDL-C, high-density lipoprotein cholesterol; T2DM, type 2 diabetes mellitus; MS, metabolic syndrome; HUA, Hyperuricemia; PCOS, Polycystic Ovary Syndrome.

**Table S9.** Original data for leptin and glucose metabolism markers by the genotypes of *LEPR* rs1137101 variant.

| **Authors, reference** | **Subjects** | **N** | | **Leptin, ng/ml** | | **Glucose, mg/dL** | | **Insulin, μU/mL** | | **HOMA-IR** | |
| --- | --- | --- | --- | --- | --- | --- | --- | --- | --- | --- | --- |
|  |  | AA | AG+GG | AA | AG+GG | AA | AG+GG | AA | AG+GG | AA | AG+GG |
| Silver et al. [1] | Overweight/obesity patients | 33 | 71 | - | - | 100.8±10.8 | 103.69±10.51 | 1.8±1.0 | 1.88±0.42 | - | - |
|  | Overweight/obesity patients | 46 | 131 | - | - | 91.8±10.8 | 92.79±19.39 | 2.1±1.9 | 2.03±1.97 | - | - |
| Chagnon et al. [2] | General subjects | 91 | 212 | 10.8±9.54 | 10.79±9.67 | - | - | - | - | - | - |
|  | General subjects | 30 | 66 | 6.0±5.48 | 10.41±4.8 | - | - | - | - | - | - |
|  | General subjects | 25 | 62 | 21.5±15 | 24.64±15.73 | - | - | - | - | - | - |
| Rosmond et al. [3] | General subjects | 71 | 198 | 6.4±4.5 | 6.05±4.20 | 86.4±27 | 81.00±12.20 | 13.8±13.2 | 12.14±9.96 | - | - |
| Ukkola et al. [4] | General subjects | 10 | 14 | 1.5±1.58 | 0.9±0.37 | - | - | 7.15±1.96 | 6.07±0.86 | - | - |
| Quinton et al. [5] | Postmenopausal subjects | 26 | 58 | 18.3±10.20 | 20.90±15.23 | - | - | - | - | - | - |
| Wauters et al. [6] | Overweight/obesity patients | 12 | 38 | 38±15.59 | 36.5±16.03 | - | - | - | - | - | - |
|  | Overweight/obesity patients | 5 | 15 | 53.7±15.43 | 32.8±17.82 | - | - | - | - | - | - |
| Wauters et al. [7] | Obesity patients | 18 | 45 | - | - | 102.6±38.16 | 100.8±36.18 | 16.80±10.96 | 22.11±10.59 | - | - |
|  | Obesity patients | 4 | 18 | - | - | 100.8±28.8 | 109.8 ±22.91 | 35.18±12.64 | 19.38±12.18 | - | - |
| Yiannakouris et al. [8] | General subjects | 52 | 66 | 7.2±6.49 | 6.22±5.04 | - | - | - | - | - | - |
| Stefan et al. [9] | Non-diabetic subjects | 124 | 144 | 23.0±26.9 | 22.86±20.38 | - | - | - | - | - | - |
|  | Non-diabetic subjects | 82 | 102 | 25.8±20.1 | 26.89±21.6 | - | - | - | - | - | - |
| Chiu et al. [10] | Glucose-tolerant subjects | 20 | 47 | - | - | 82.44±6.66 | 84.66±6.12 | 7.9±2.45 | 8.62±2.51 | - | - |
| Guizar-Mendoza et al. [11] | Obese patients/lean subjects | 41 | 62 | 38.7±23.5 | 30.67±23.53 | - | - | 1.55±1.25 | 1.36±1.12 | - | - |
| Mendez-Sanchez et al. [12] | Gallstones disease patients | 37 | 60 | 12.02±7.66 | 14.33±9.04 | 107.29±72.32 | 96.10±18.79 | 0.87±0.47 | 1.05±0.65 | 1.7±1.89 | 1.75±1.23 |
| Van der Vleuten et al. [13] | General subjects | 171 | 307 | - | - | - | - | - | - | 2.04±1.39 | 2.01±1.46 |
|  | General subjects | 77 | 138 | 3.9±3.97 | 4.3±3.86 | - | - | - | - | - | - |
|  | General subjects | 94 | 169 | 18.7±14.65 | 19.1±16.13 | - | - | - | - | - | - |
| Masuo et al. [14] | Lean subjects | 35 | 5 | 4.02±3.25 | 7.70±3.45 | - | - | - | - | - | - |
|  | Obese patients/lean subjects | 63 | 26 | 8.15±3.77 | 16.01±6.67 | - | - | - | - | - | - |
| Ben et al. [17] | Control subjects | 113 | 189 | 6.5±4.9 | 5.89±4.14 | - | - | - | - | - | - |
|  | Obese patients | 133 | 258 | 21.9±11.9 | 19.18±8.85 | - | - | - | - | - | - |
| Constantin et al. [18] | Overweight/obesity patients/control subjects | 62 | 140 | 14.9±6.2 | 15.7±5.9 | 133.6±67.0 | 148.7±66.3 | 8.95±3.6 | 8.5±4.0 | 3.0±2.3 | 3.3±2.7 |
| Riestra et al. [19] | General subjects | 138 | 277 | 16.5±10.8 | 16.00±9.87 | - | - | - | - | - | - |
|  | General subjects | 120 | 254 | 7.4±9.4 | 5.63±7.52 | - | - | - | - | - | - |
| Saukko et al. [20] | General subjects | 81 | 442 | - | - | 81±12.24 | 79.20±8.1 | - | - | - | - |
| Labayen et al. [21] | General subjects | 269 | 487 | 20.1±23.0 | 20.72±23.91 | - | - | - | - | - | - |
| Sun et al. [22] | T2DM patients | 2 | 201 | - | - | 235.8±13.1 | 178.42±67.36 | - | - | - | - |
|  | Control subjects | 10 | 286 | - | - | 88.2±1.8 | 78.4±18.09 | - | - | - | - |
| Angel-Chavez et al. [23] | Overweight/obesity patients/control subjects | 37 | 91 | - | - | 90.61±11.8 | 87.69±11.1 | - | - | - | - |
| Boumaiza et al. [24] | Overweight/obesity patients/control subjects | 136 | 193 | - | - | 108.18±38.34 | 122.03±59.85 | 6.45±4.41 | 8.71±6.98 | 1.57±1.41 | 2.2±1.98 |
| Huuskonen et al. [25] | General subjects | 125 | 588 | 4.161±4.378 | 3.67±3.70 | - | - | - | - | - | - |
| Jackson et al. [26] | The postprandial lipaemic response subjects | 35 | 74 | - | - | 88.74±8.46 | 87.50±8.85 | 6.46±4.76 | 4.72±6.36 | 1.5±1.18 | 1.07±1.57 |
|  | The postprandial lipaemic response subjects | 36 | 86 | - | - | 98.10±14.04 | 95.25±19.14 | 7.22±4.74 | 7.79±4.85 | 1.9±1.20 | 1.94±1.65 |
| Becer et al. [27] | Control subjects | 40 | 50 | 8.05±4.98 | 8.44±3.22 | 90.75±7.69 | 88.92±7.24 | - | - | 1.97±0.77 | 1.85±0.57 |
|  | Obese patients | 38 | 72 | 20.39±11.54 | 26.62±14.61 | 100.71±14.92 | 104.61±26.89 | - | - | 3.88±2.31 | 4.95±4.55 |
| Zandona et al. [28] | General subjects | 97 | 216 | - | - | 70.7±7.4 | 72.89±6.4 | - | - | - | - |
| Zheng et al. [29] | Hypertensive left ventricular hypertrophy patients | 10 | 180 | 1.23±0.37 | 0.93±0.27 | 96.6±5.2 | 98.69±5.37 | - | - | - | - |
| Aijala et al. [30] | Hypertensive patients/control subjects | 167 | 867 | 11.4±9.4 | 10.43±7.96 | - | - | - | - | - | - |
| Fan et al. [32] | Overweight/obesity patients/control subjects | 28 | 380 | 30.06±36.68 | 28.60±36.34 | - | - | - | - | - | - |
| Murakami et al. [33] | General subjects | 5 | 551 | - | - | 91.4±10.1 | 90.42±12.89 | - | - | - | - |
| Chen et al. [34] | MS patients | 42 | 125 | - | - | 134.28±14.04 | 118.17±18.49 | 21.82±6.91 | 18.13±6.74 | 7.72±1.67 | 5.05±2.11 |
| Wu et al. [35] | MS patients/control subjects | 44 | 2038 | 9.85±8.16 | 11.68±13.83 | 128.52±51.66 | 121.72±47.65 | 0.89±1.16 | 1.21±0.77 | 1. 84±2.60 | 2.23±1.75 |
| Martins et al. [36] | Control subjects | 38 | 107 | 16.1±12.17 | 15.5±10.09 | - | - | - | - | - | - |
| Wu et al. [37] | Hypertension patients/control subjects | 9 | 513 | 4.08±5.61 | 2.57±4.62 | 83.88±8.1 | 86.76±8.67 | 5.31±2.22 | 5.62±3.71 | 1.08±0.40 | 1.2±0.77 |
| Zayani et al. [38] | General subjects | 585 | 536 | - | - | 105.84±39.96 | 102.06±33.11 | - | - | - | - |
| Daghestani et al. [39] | Control subjects | 42 | 20 | 11.32±3.05 | 12.58±5.03 | 81.36±7 | 81.54±8.2 | 7.41±2.59 | 7.92±2.57 | 0.84±0.58 | 1.03±0.28 |
|  | Obese patients | 39 | 23 | 33.68±16.24 | 51.32±22.9 | 86.76±7.87 | 91.08±9.74 | 11.85±6.18 | 16.3±4.63 | 1.52±0.81 | 2.1±0.59 |
| Daghestani et al. [40] | PCOS patients | 85 | 45 | 28.2±19.0 | 32.86±49.71 | 91.62±12.24 | 91.07±21.55 | 15.91±12.61 | 14.67±19.48 | 3.61±19.8 | 3.34±17.14 |
|  | Control subjects | 82 | 40 | 22.2±19.7 | 33.85±62.06 | 83.88±9.9 | 86.63±23.77 | 9.58±6.39 | 12.49±13.82 | 2.04±1.43 | 2.75±3.31 |
| Bains et al. [42] | General subjects | 200 | 200 | - | - | 84.95±11.61 | 86.42±13.08 | - | - | - | - |
| Dieguez-Campa et al. [43] | Obese patients | 20 | 36 | 24.96±18.2 | 26.79±19.7 | - | - | - | - | - | - |
|  | Control subjects | 33 | 70 | 13.73±14.94 | 16.00±11.94 | - | - | - | - | - | - |
| Illangasekera et al. [44] | Overweight/obesitypatients/control subjects | 107 | 423 | - | - | 95.4±45 | 98.23±60.31 | - | - | - | - |
| Ali et al. [45] | Obese patients | 49 | 61 | 15.3±3.4 | 15.5±3.1 | 127.7±35.7 | 132.2±35.5 | 14.9±5.5 | 16.0±6.5 | 5.1 ± 3.1 | 5.7 ± 3.6 |
| Hu et al. [46] | T2DM/HUA | 15 | 65 | - | - | 182.16±43.2 | 176.19±40.44 | - | - | - | - |
| Mohanraj et al. [47] | General subjects | 35 | 28 | 2.93±0.69 | 3.00±0.75 | - | - | - | - | - | - |
| Sanchez-Murguia et al. [48] | General subjects | 37 | 95 | 12.4±5.5 | 10.5±6.6 | - | - | 7.5±3.7 | 7.2±4.5 | - | - |

LEPR, leptin receptor; HOMA-IR, homeostasis model assessment of insulin resistance; T2DM, type 2 diabetes mellitus; MS, metabolic syndrome; HUA, hyperuricemia; PCOS, polycystic ovary Syndrome.

**Table S10.** Original data for lipid metabolism markers by the genotypes of *LEPR* rs1137101 variant.

| **Authors, reference** | **Subjects** | **N** | | **Triglycerides, mg/dL** | | **TC, mg/dL** | | **LDL-C, mg/dL** | | **HDL-C, mg/dL** | |
| --- | --- | --- | --- | --- | --- | --- | --- | --- | --- | --- | --- |
|  |  | AA | AG+GG | AA | AG+GG | AA | AG+GG | AA | AG+GG | AA | AG+GG |
| Rosmond et al. [3] | General subjects | 71 | 198 | 185.94±97.4 | 152.94±92.85 | 239.75±42.54 | 238.35±41.12 | 162.41±42.54 | 158.55±38.67 | 42.54±11.60 | 50.27±14.15 |
| Ukkola et al. [4] | General subjects | 10 | 14 | 115.11±56 | 88.55±33.11 | 181.75±24.44 | 166.28±28.92 | - | - | 46.40±0.0 | 46.40±14.48 |
| Chiu et al. [10] | Glucose tolerant subjects | 20 | 47 | 83±41.67 | 80.60±40.54 | 144±22.44 | 162.62±28.15 | 81±22.44 | 97.68±25.28 | 47±11.75 | 49.62±11.86 |
| Mendez-Sanchez et al. [12] | Gallstones disease patients | 37 | 60 | 161.86±79.88 | 190.88±141.9 | 198.7±42.81 | 211.00±38.01 | 127.29±32.99 | 136.83±43.62 | 38.91±12.15 | 40.62±10.18 |
| Van der Vleuten et al. [13] | General subjects | 171 | 307 | 89.43±44.27 | 96.51±46.93 | 186.39±40.99 | 192.19±42.92 | - | - | 49.50±13.92 | 46.79±13.92 |
| Zhao et al. [14] | Hypertension/obesity patients | 82 | 78 | 193.03±61.98 | 191.26±53.13 | 166.67±62.26 | 165.51±58.39 | 134.96±44.47 | 130.70±45.24 | 48.34±12.76 | 41.76±8.12 |
|  | Hypertension/obesity patients | 38 | 58 | 169.12±61.98 | 169.12±79.69 | 169.76±62.26 | 166.67±66.9 | 131.86±50.66 | 131.86±42.54 | 48.72±13.53 | 46.79±14.69 |
|  | Control subjects | 72 | 180 | 102.71±26.56 | 115.99±44.27 | 135.73±6.19 | 137.67±7.35 | 97.06±4.25 | 83.53±4.64 | 46.79±6.19 | 46.79±8.12 |
| Zhao et al. [16] | Obesity/T2DM patients | 88 | 73 | 193.03±61.98 | 191.26±53.13 | 166.67±62.26 | 165.51±58.39 | 134.96±44.47 | 130.70±45.24 | 48.34±12.76 | 41.76±8.12 |
|  | Obesity/T2DM patients | 40 | 53 | 169.12±61.98 | 169.12±79.69 | 169.76±62.26 | 166.67±66.90 | 131.86±50.66 | 131.86±42.54 | 48.72±13.53 | 46.79±14.69 |
| Ben et al. [17] | control subjects | 113 | 189 | 106.25±88.55 | 92.81±48.25 | 181.75±38.67 | 174.02±31.89 | 108.28±34.80 | 100.54±29.91 | 50.27±11.60 | 50.27±11.57 |
|  | Obese patients | 133 | 258 | 132.82±70.84 | 134.88±70.80 | 185.62±38.67 | 186.52±33.94 | 112.14±34.80 | 113.03±30.13 | 42.54±7.73 | 45.50±7.89 |
| Constantin et al. [18] | Overweight/obesity patients/control subjects | 62 | 140 | 125.3±94.9 | 135.9±79.1 | 219.1±46.0 | 211.50±52.1 | 138.3±41.5 | 131.5±38.5 | 52.5±14.2 | 46.3±14.0 |
| Saukko et al. [20] | General subjects | 81 | 442 | 97.40±60.21 | 111.32±20.82 | 208.82±34.81 | 214.34±26.35 | 127.61±34.80 | 133.13±21.72 | 54.14±8.89 | 50.27±8.88 |
| Angel-Chavez et al. [23] | Overweight/obesity patients/control subjects | 37 | 91 | 109.52±48.0 | 120.88±47.2 | 148.55±44 | 144.59±39.4 | 99.30±21.3 | 100.31±24.3 | 38.71±10.1 | 38.48±9.3 |
| Boumaiza et al. [24] | Overweight/obesity patients/control subjects | 136 | 193 | 91.2±47.81 | 104.53±49.51 | 174.02±37.12 | 184.19±45.11 | - | - | 50.27±15.85 | 44.26±15.16 |
| Jackson et al. [26] | The postprandial lipaemic response subjects | 35 | 74 | - | - | 210.36±39.06 | 216.52±39.43 | 126.45±36.74 | 134.64±40.13 | 62.65±15.85 | 62.49±15.18 |
|  | The postprandial lipaemic response subjects | 36 | 86 | - | - | 226.22±44.08 | 219.57±36.58 | 146.56±41.76 | 144.52±32.42 | 42.92±9.28 | 44.40±10.15 |
| Becer et al. [27] | Control subjects | 40 | 50 | 99.83±43.74 | 109.88±36.93 | 189.90±23.99 | 214.6±37.41 | 121.13±26.27 | 126.04±29.7 | 54.53±10.08 | 55.9±8.83 |
|  | Obese patients | 38 | 72 | 157.76±70.18 | 171.83±89.66 | 224.55±46.54 | 238.99±30.60 | 144.55±40.02 | 144.54±27.89 | 47.53±11.84 | 48±9.35 |
| Zheng et al. [29] | Hypertensive left ventricular hypertrophy patients | 10 | 180 | 144.6±9.6 | 146.29±11.05 | 193.4±21.6 | 204.97±20.91 | 126.7±14.9 | 118.44±16.12 | 48.1±8.2 | 46.7±10.81 |
| Aijala et al. [30] | Hypertensive patients/control subjects | 167 | 867 | - | - | - | - | 135.35±34.8 | 135.35±36.44 | - | - |
| Al-Azzam et al. [31] | Diabetic patients | 216 | 162 | 189.49±155.84 | 210.29±205.84 | 191.8±47.18 | 195.91±56.56 | 119.49±39.06 | 125.25±46.15 | 46.79±27.84 | 44.58±19.56 |
| Murakami et al. [33] | General subjects | 5 | 551 | 69.0±31.0 | 90.43±57.18 | - | - | - | - | 72.6±20.4 | 64.78±15.88 |
| Chen et al. [34] | MS patients | 42 | 125 | 277.15±54.9 | 197.9±65.72 | 272.24±27.46 | 228.36±26.62 | 170.53±19.72 | 126.63±16.45 | 26.68±10.44 | 38.05±17.35 |
| Wu et al. [35] | MS patients/control subjects | 44 | 2038 | 202.77±100.06 | 162.7±88.58 | 178.66±34.80 | 178.56±39.83 | 108.28±22.82 | 99.09±33.35 | 37.12±8.12 | 44.76±10.49 |
| Wu et al. [37] | Hypertension patients/control subjects | 9 | 513 | 97.40±36.30 | 92.13±53.45 | 197.60±56.46 | 168.49 ±31.78 | 119.10±52.20 | 93.38 ± 26.17 | 57.23±12.37 | 55.3±11.65 |
| Zayani et al. [38] | General subjects | 585 | 536 | - | - | 187.94±38.67 | 192±39.07 | 123.36±31.71 | 128.16±32.1 | 46.02±15.85 | 43.92±12.76 |
| Daghestani et al. [39] | Control subjects | 42 | 20 | 59.33±22.94 | 62.87±19.09 | 130.7±2.71 | 135.54±5.34 | 50.66±15.04 | 49.97±12.18 | 56.07±12.25 | 54.81±12.23 |
|  | Obese patients | 39 | 23 | 87.66±38.72 | 99.74±38.8 | 146.56±3.48 | 153.6±8.66 | 80.43±21.73 | 82.55±25.56 | 44.08±12.05 | 41.41±10.38 |
| Daghestani et al. [40] | PCOS patients | 85 | 45 | 92.97±50.47 | 114.24±104.93 | 174.02±4.41 | 135.54±5.34 | 98.61±39.83 | 99.96±66.88 | 43.31±149.27 | 39.14±23.13 |
|  | Control subjects | 82 | 40 | 73.49±38.96 | 82.96±86.55 | 138.05±25.52 | 145.41±35.37 | 65.35±29.78 | 67.86±59.65 | 49.88±12.76 | 47.31±27.92 |
| Zheng et al. [41] | Control subjects | 421 | 106 | 131.05±641.95 | 126.75±280.55 | 186.78±37.90 | 188.35±37.69 | 105.18±29.00 | 107.44±29.41 | 48.72±11.60 | 48.34±10.84 |
|  | Overweight/obesity patients | 244 | 66 | 183.29±733.15 | 208.97±300.01 | 196.44±39.44 | 194.07±33.63 | 110.21±33.26 | 110.92±27.25 | 42.54±10.83 | 43.92±10.15 |
| Bains et al. [42] | General subjects | 200 | 200 | 137.42±42.81 | 142.94±45.45 | 173.33±36.05 | 169.95±33.09 | 101.15±34.12 | 95.56±29.83 | 44.69±13.12 | 45.80±12.94 |
| Illangasekera et al. [44] | Overweight/obesity patients/control subjects | 107 | 423 | 292.2±141.67 | 301.05±182.73 | 212.69±34.8 | 224.29±61.05 | 135.35±34.8 | 149.71±92.61 | 50.27±11.6 | 56.63±47.04 |
| Ali et al. [45] | Obese patients | 49 | 61 | 196.2±33.9 | 200.8±37.3 | 209.7±27.3 | 212.9±24.1 | 117.6±21.1 | 118.5±19.3 | 52.9±8.8 | 54.1±7.3 |

LEPR, leptin receptor; TC, total cholesterol; LDL-C, low-density lipoprotein cholesterol; HDL-C, high-density lipoprotein cholesterol; T2DM, type 2 diabetes mellitus; MS, metabolic syndrome; HUA, hyperuricemia; PCOS, polycystic Ovary Syndrome.

**Table S11.** Characteristics of the studies included in the meta-analysis for *LEPR* rs1805094 variant.

| **Authors, reference** | **Publication year** | **Ethnicity** | **Gender** | **Subjects** | **Age**  **(Mean**±SD **or age range**) | **Outcomes** |
| --- | --- | --- | --- | --- | --- | --- |
| Rosmond et al. [1] | 2000 | Caucasian | M | General subjects | 51 | Leptin/glucose/insulin/triglycerides/TC/LDL-C/HDL-C |
| Wauters et al. [2] | 2001 | Caucasian | F | Overweight/obesity patients | 39±10.98 | Leptin |
| Wauters et al. [3] | 2001 | Caucasian | F | T2DM patients/impaired glucose tolerance subjects | 18-60 | Glucose/insulin |
| De Luis Roman et al. [4] | 2006 | Caucasian | M/F | Overweight/obesity patients | 45.7±16.6 | Leptin/glucose/insulin/HOMA-IR/TC/LDL-C/HDL-C |
| De Luis et al. [5] | 2008 | Caucasian | M/F | Overweight/obesity patients | 46.7±13.6 | Leptin/glucose/insulin/HOME-IR/triglycerides/TC/LDL-C/HDL-C |
| De Luis et al. [6] | 2008 | Caucasian | M/F | Overweight/obesity/T2DM patients | 56.9±11.2 | Leptin/glucose/insulin/HOMA-IR/triglycerides/TC/LDL-C/HDL-C |
| De Luis et al. [7] | 2008 | Caucasian | M/F | Overweight/obesity patients | 43.6±16.6 | Leptin/glucose/insulin/HOMA-IR/triglycerides/TC/LDL-C/HDL-C |
| Masuo et al. [8] | 2008 | Caucasian | M | Overweight/obesity patients/control subjects | 23-59 | Leptin |
| Perez-Castrillon et al. [9] | 2009 | Caucasian | M/F | Acute coronary syndrome patients | 61±10 | Triglycerides/TC |
| De Luis et al. [10] | 2010 | Caucasian | M/F | Overweight/obesity patients | 43.37±7.41 | Glucose/triglycerides/TC/LDL-C/HDL-C |
| Murugesan et al. [11] | 2010 | Asian | M/F | T2DM patients/control subjects | 41-59 | Leptin/insulin |
| Riestra et al. [12] | 2010 | Caucasian | M/F | General subjects | 12-16 | Leptin |
| Labayen et al. [13] | 2011 | Caucasian | M/F | General subjects | 12.5-17.5 | Leptin |
| Angel-Chavez et al. [14] | 2012 | Latin American | M/F | Overweight/obesity patients/control subjects | 6-17 | Glucose/triglycerides/LDL-C/HDL-C |
| Huuskonen et al. [15] | 2012 | Caucasian | M | General subjects | 25±5 | Leptin |
| De Luis et al. [16] | 2013 | Caucasian | M/F | Overweight/obesity patients | 48.3±14.2 | Leptin/glucose/insulin/HOMA-IR/TC/LDL-C/HDL-C |
| De Luis et al. [17] | 2015 | Caucasian | M/F | Overweight/obesity patients | 37.3±6.1 | Leptin/glucose/insulin/HOMA-IR/triglycerides/TC/LDL-C/HDL-C |
| Tang et al. [18] | 2017 | Asian | M/F | General subjects | 20 | Insulin/triglycerides/TC/LDL-C/HDL-C |
| Foucan et al. [19] | 2019 | Latin American | M/F | General subjects | 46±12 | Leptin |
| Jang et al. [20] | 2020 | Asian | F | Overweight/obesity patients | 44.42±8.75 | Leptin/glucose/insulin/HOMA-IR/triglycerides/TC/LDL-C/HDL-C |

LEPR, leptin receptor; SD, standard deviation; M, male; F, female; HOMA-IR, homeostasis model assessment of insulin resistance; TC, total cholesterol; LDL-C, low-density lipoprotein cholesterol; HDL-C, high-density lipoprotein cholesterol; T2DM, type 2 diabetes mellitus.

**Table S12.** Original data for leptin and glucose metabolism markers by the genotypes of *LEPR* rs1805094 variant.

| **Authors, reference** | **Subjects** | **N** | | **Leptin, ng/ml** | | **glucose, mg/dL** | | **Insulin, μU/mL** | | **HOMA-IR** | |
| --- | --- | --- | --- | --- | --- | --- | --- | --- | --- | --- | --- |
|  |  | GG | GC+CC | GG | GC+CC | GG | GC+CC | GG | GC+CC | GG | GC+CC |
| Rosmond et al. [1] | General subjects | 197 | 72 | 5.8±4.1 | 7.0±4.6 | 81±16.2 | 84.00±20.49 | 12.3±10.3 | 13.43±12.42 | - | - |
| Wauters et al. [2] | Overweight/obesity patients | 32 | 18 | 37.1±16.4 | 35.9±15.7 | - | - | - | - | - | - |
|  | Overweight/obesity patients | 12 | 8 | 33.8±17.32 | 45.7±17.54 | - | - | - | - | - | - |
| Wauters et al. [3] | T2DM patients/impaired glucose tolerance subjects | 44 | 19 | - | - | 108±35.82 | 86.4±31.38 | 21.39±10.48 | 18.52±11.25 | - | - |
|  | Impaired glucose tolerance/T2DM patients | 15 | 6 | - | - | 100.8±27.89 | 124.2±26.45 | 18.09±12.24 | 32.3±12.66 | - | - |
| De Luis Roman et al. [4] | Overweight/obesity patients | 36 | 31 | 53.7±31.5 | 67±24 | 97.5±21.2 | 104.8±22 | 14.5±7.5 | 12.8±8.1 | 2.51±1.4 | 2.1±1.2 |
| De Luis et al. [5] | Overweight/obesity patients | 22 | 14 | 135.5±65 | 160±66 | 95.8±9.5 | 104.6±13 | 20.2±12 | 17.5±6.2 | 2.8±2.1 | 3.2±1.6 |
|  | Overweight/obesity patients | 30 | 12 | 133.1±64 | 173±61 | 100.3±20.8 | 99.2±22 | 13.5±7.7 | 14.6±18.2 | 2.7±2.1 | 2.5±1.6 |
| De Luis et al. [6] | Overweight/obesity /T2DM patients | 36 | 23 | 76.6±73 | 123.4±85 | 130.5±27 | 134.3±24 | 22.7±21 | 26.3±22 | 7.6±7.3 | 9.1±9.3 |
| De Luis et al. [7] | Overweight/obesity patients | 49 | 18 | 39.3±23 | 63.5±28 | 99.9±23 | 103.8±25 | 18.1±10.7 | 32.1±25 | 2.8±1.7 | 5.6±4.8 |
|  | Overweight/obesity patients | 94 | 70 | 121.3±81 | 136.8±88 | 98.7±18.4 | 100.5±20.7 | 15.6±9.2 | 15.9±8.9 | 2.4±1.5 | 2.4±1.7 |
| Masuo et al. [8] | Overweight/obesity patients | 32 | 57 | 8.85±3.95 | 11.35±6.01 | - | - | - | - | - | - |
|  | Control subjects | 21 | 19 | 4.10±2.78 | 4.92±2.22 | - | - | - | - | - | - |
| Perez-Castrillon et al. [9] | Acute coronary syndrome | 42 | 20 | - | - | - | - | - | - | - | - |
| De Luis et al. [10] | Overweight/obesity patients | 32 | 8 | - | - | 104.6±18 | 114.6±25 | - | - | - | - |
| Murugesan et al. [11] | T2DM patients | 175 | 125 | 29.54±12.14 | 27.59±16.68 | - | - | 22.97±7.2 | 23.00±9.77 | - | - |
|  | Control subjects | 175 | 125 | 23.44±11.36 | 19.19±12.09 | - | - | 20.61±6.4 | 17.18±6.26 | - | - |
| Riestra et al. [12] | General subjects | 224 | 119 | 5.7±7.4 | 7.23±9.55 | - | - | - | - | - | - |
|  | General subjects | 254 | 129 | 15.7±9.8 | 16.63±10.59 | - | - | - | - | - | - |
| Labayen et al. [13] | General subjects | 496 | 223 | 19.0±22.3 | 23.87±25.91 | - | - | - | - | - | - |
| Angel-Chavez et al. [14] | Control subjects | 38 | 14 | - | - | 87.33±10.0 | 89.67±13.4 | - | - | - | - |
|  | Overweight/obesity patients | 64 | 12 | - | - | 88.77±12.1 | 85.62±8.9 | - | - | - | - |
| Huuskonen et al. [15] | General subjects | 549 | 164 | 3.75±3.68 | 3.78±4.30 | - | - | - | - | - | - |
| De Luis et al. [16] | Overweight/obesity patients | 88 | 38 | 30.1±24.5 | 18.9±11.8 | 91.3±11.2 | 94.6±10.1 | 13.6±8.5 | 10.6±6.1 | 3.4±2.4 | 2.6±1.2 |
| De Luis et al. [17] | Overweight/obesity patients | 88 | 44 | 32.3±25.5 | 24.9±14.8 | 95.2±10.2 | 94.2±9.1 | 12.9±8.7 | 13.4±6.8 | 3.3±1.8 | 3.7±2.2 |
| Tang et al. [18] | General subjects | 24 | 3 | - | - | - | - | 4.5±2.6 | 12.4±10.8 | - | - |
|  | General subjects | 26 | 3 | - | - | - | - | 4.8±3.9 | 2.4±0.7 | - | - |
| Foucan et al. [19] | General subjects | 240 | 135 | 12.7±14.0 | 17.4±20.4 | - | - | - | - | - | - |
| Jang et al. [20] | Overweight/obesity patients | 151 | 26 | 22.9±10.3 | 18.1±5.94 | 98.8±8.77 | 86.1±6.90 | 12.5±4.93 | 10.7±3.13 | 3.07±1.31 | 2.27±0.72 |

LEPR, leptin receptor; HOMA-IR, homeostasis model assessment of insulin resistance; TC, total cholesterol; LDL-C, low-density lipoprotein cholesterol; HDL-C, high-density lipoprotein cholesterol; T2DM, type 2 diabetes mellitus.

**Table S13.** Original data for lipid metabolism markers by the genotypes of *LEPR* rs1805094 variant.

| **Authors, reference** | **Subjects** | **N** | | **Triglycerides, mg/dL** | | **TC, mg/dL** | | **LDL-C, mg/dL** | | **HDL-C, mg/dL** | |
| --- | --- | --- | --- | --- | --- | --- | --- | --- | --- | --- | --- |
|  |  | GG | GC+CC | GG | GC+CC | GG | GC+CC | GG | GC+CC | GG | GC+CC |
| Rosmond et al. [1] | General subjects | 197 | 72 | 159.38±97.40 | 164.3±78.18 | 239.75±42.54 | 239.75±41.23 | 158.55±38.67 | 159.41±41.31 | 50.27±11.60 | 46.4±11.52 |
| De Luis Roman et al. [4] | Overweight/obesity patients | 36 | 31 | 129.6±57.8 | 109.6±44 | 211.6±49 | 212.1±39.9 | 125.5±58 | 139.5±32.3 | 57.3±19.9 | 53.9±11.4 |
| De Luis et al. [5] | Overweight/obesity patients | 22 | 14 | 148.3±45 | 124.8±62.4 | 205.5±36 | 212.4±37 | 117.9±38 | 130.8±49 | 54.1±10.7 | 53.7±10.6 |
|  | Overweight/obesity patients | 30 | 12 | 131.2±41.8 | 121.88±62.4 | 189.8±28 | 212.4±48.4 | 117.7±29 | 124.8±49 | 55.7±19.2 | 56.9±12.6 |
| De Luis et al. [6] | Overweight/obesity/T2DM patients | 36 | 23 | 152.6±61 | 163±73 | 211.8±38 | 220±44 | 143.4±36 | 140.6±39 | 51.4±10 | 52.7±11 |
| De Luis et al. [7] | Overweight/obesity patients | 49 | 18 | 146.8±68 | 140.4±58.5 | 206.5±39 | 191.8±26.5 | 130.3±35 | 124.1±23.5 | 57.3±19.9 | 53.9±11.4 |
|  | Overweight/obesity patients | 94 | 70 | 115±56 | 115.5±50 | 203.2±46 | 212±41.6 | 122.5±46 | 128.8±38 | 54.5±54.5 | 55.3±11.8 |
| Perez-Castrillon et al. [9] | Acute coronary syndrome | 42 | 20 | 142±70 | 174±129 | 173±41 | 200±56 | - | - | - | - |
| De Luis et al. [10] | Overweight/obesity patients | 32 | 8 | 140.5±66 | 123.7±61 | 190.9±35 | 195.3±37 | 89.8±28 | 119±35 | 50±15 | 50.4±7.4 |
| Angel-Chavez et al. [14] | Control subjects | 38 | 14 | 118.73±42.8 | 92.5±40.9 | - | - | 96.92±22.1 | 91.35±21.6 | 39.02±9.7 | 43.41±10.0 |
|  | Overweight/obesity patients | 64 | 12 | 120.06±50.7 | 110.22±31.6 | - | - | 101.60±23.2 | 102.15±31.6 | 36.82±9.2 | 45.44±12.0 |
| De Luis et al. [16] | Overweight/obesity patients | 88 | 38 | 124.7±55.8 | 114.4±42.7 | 209.1±35.4 | 204.4±34.9 | 129.4±32.1 | 130.9±32.1 | 54.3±17.9 | 51.2±11.4 |
| De Luis et al. [17] | Overweight/obesity patients | 88 | 44 | 136.2±71.8 | 128.2±42.7 | 207.6±47.4 | 208.3±45.9 | 133.9±42.1 | 132.5±37.1 | 51.4±11.9 | 49.1±12.4 |
| Tang et al. [18] | General subjects | 24 | 3 | 78.3±32.0 | 107.6±73.3 | 147.0±24.5 | 150.9±14.6 | 66.1±23.5 | 64.8±16.3 | 51.7±10.2 | 42.8±14.3 |
|  | General subjects | 26 | 3 | 66.0±16.6 | 62.6±11.5 | 160.3±28.2 | 156.9±11.1 | 71.2±22.0 | 65.1±5.7 | 60.6±10.2 | 57.2±9.5 |
| Jang et al. [20] | Overweight/obesity patients | 151 | 26 | 125.4±60.2 | 98.1±43.0 | 203.5±36.7 | 192.2±40.8 | 129.9±33.9 | 113.0±36.0 | 53.5±12.5 | 59.6±12.6 |

LEPR, leptin receptor; HOMA-IR, homeostasis model assessment of insulin resistance; TC, total cholesterol; LDL-C, low-density lipoprotein cholesterol; HDL-C, high-density lipoprotein cholesterol; T2DM, type 2 diabetes mellitus.

**Table S14.** Meta-analyses between *LEPR* rs1137100 variant and leptin and glucose metabolism markers.

| Groups or subgroups | Comparisons (Subjects) | SMD (95% CI) | *P*_Heterogeneity_ | *P*_SMD_ |
| --- | --- | --- | --- | --- |
| Leptin (GG + AG vs. AA) |  |  |  |  |
| All subjects | 8 (5168) | -0.15 (-0.33, 0.01 ) | <0.001 | 0.07 |
| Caucasians | 6 (2564) | -0.18 (-0.38, 0.02) | <0.001 | 0.08 |
| Overweight/obesity patients | 3 (446) | -0.36 (-1.05, 0.32 ) | <0.001 | 0.29 |
| Glucose (GG + AG vs. AA) |  |  |  |  |
| All subjects | 8 (4148) | 0.02 (-0.07, 0.11) | 0.81 | 0.67 |
| Caucasians | 6 (1544) | 0.03 (-0.07, 0.13) | 0.81 | 0.54 |
| Impaired glucose tolerance subjects | 3 (592) | 0.12 (-0.05, 0.28) | 0.84 | 0.16 |
| Overweight/obesity patients | 3 (251) | 0.11 (-0.15, 0.36) | 0.76 | 0.42 |
| Insulin (GG + AG vs. AA) |  |  |  |  |
| All subjects | 7 (3629) | 0.03 (-0.14, 0.20) | 0.09 | 0.72 |
| Caucasians | 5 (1025) | 0.02 (-0.22, 0.25) | 0.04 | 0.88 |
| Impaired glucose tolerance subjects | 3 (592) | -0.12 (-0.65, 0.41) | 0.03 | 0.66 |
| Overweight/obesity patients | 3 (251) | -0.05 (-0.68, 0.58) | 0.02 | 0.87 |
| HOMA-IR (GG + AG vs. AA) |  |  |  |  |
| All subjects | 5 (3218) | 0.03 (-0.10, 0.15) | 0.57 | 0.69 |

*LEPR*, leptin receptor gene; SMD, standardized mean difference; 95% CI, 95% confidence interval; HOMA-IR, homeostasis model assessment of insulin resistance.

**Table S15.** Meta-analyses between *LEPR* rs1137100 variant and lipid-metabolism markers.

| Groups or subgroups | Comparisons (Subjects) | SMD (95% CI) | *P*_Heterogeneity_ | *P*_SMD_ |
| --- | --- | --- | --- | --- |
| Triglycerides (GG + AG vs. AA) |  |  |  |  |
| All subjects | 6 (3692) | -0.08 (-0.19, 0.03) | 0.37 | 0.16 |
| Adults | 5 (3556) | -0.10 (-0.22, 0.02) | 0.33 | 0.11 |
| Asians | 3 (2740) | -0.11 (-0.29, 0.08) | 0.47 | 0.25 |
| Caucasians | 3 (952) | -0.09 (-0.28, 0.10) | 0.15 | 0.37 |
| TC (GG + AG vs. AA) |  |  |  |  |
| All subjects | 7 (3872) | 0.08 (-0.24, 0.39 ) | < 0.001 | 0.63 |
| Adults | 6 (3736) | 0.01 (-0.33, 0.35) | < 0.001 | 0.96 |
| Asians | 4 (2920) | 0.42 (-0.31, 1.14) | < 0.001 | 0.26 |
| Caucasians | 3 (952) | -0.09 (-0.32, 0.14) | 0.07 | 0.44 |
| LDL-C (GG + AG vs. AA) |  |  |  |  |
| All subjects | 7 (4720) | -0.01 (-0.17, 0.15 ) | 0.01 | 0.90 |
| Adults | 6 (4584) | -0.05 (-0.20, 0.10) | 0.02 | 0.51 |
| Caucasians | 4 (1980) | 0.02 (-0.11, 0.15) | 0.18 | 0.75 |
| Asians | 3 (2740) | -0.09 (-0.59, 0.42) | 0.002 | 0.73 |
| HDL-C (GG + AG vs. AA) |  |  |  |  |
| All subjects | 6 (3692) | 0.20 (-0.02, 0.42 ) | 0.003 | 0.07 |
| Adults | 5 (3556) | 0.15 (-0.09, 0.39) | 0.003 | 0.22 |
| Asians | 3 (2740) | 0.21 (-0.19, 0.62) | 0.02 | 0.30 |

*LEPR*, leptin receptor gene; SMD, standardized mean difference; 95% CI, 95% confidence interval; TC, total cholesterol; LDL-C, low-density lipoprotein cholesterol; HDL-C, high-density lipoprotein cholesterol.

**Table S16.** Meta-analyses between *LEPR* rs1805094 variant and leptin and glucose metabolism markers.

| Groups or subgroups | Comparisons (Subjects) | SMD (95% CI) | *P*_Heterogeneity_ | *P*_SMD_ |
| --- | --- | --- | --- | --- |
| Leptin (CC + CG vs. GG) |  |  |  |  |
| All subjects | 21 (4471) | 0.12 (-0.02, 0.26) | < 0.001 | 0.08 |
| Females | 5 (794) | 0.02 (-0.26, 0.29) | 0.06 | 0.91 |
| Males | 6 (1521) | 0.29 (0.07, 0.51) | 0.02 | < 0.01 |
| Adults | 18 (3026) | 0.13 (-0.05, 0.30) | < 0.001 | 0.16 |
| Children/adolescents | 3 (1445) | 0.17 (0.06, 0.28) | 0.69 | < 0.01 |
| Caucasians | 17 (3319) | 0.20 (0.06, 0.33) | < 0.001 | < 0.01 |
| Asians | 3 (777) | -0.29 (-0.48, -0.10) | 0.23 | < 0.01 |
| Overweight/obesity patients | 12 (1029) | 0.20 (-0.08, 0.49) | < 0.001 | 0.16 |
| General/control subjects | 8 (3142) | 0.11 (-0.04, 0.26) | 0.001 | 0.15 |
| Glucose (CC + CG vs. GG) |  |  |  |  |
| All subjects | 15 (1391) | 0.03 (-0.24, 0.31) | < 0.001 | 0.81 |
| Females | 4 (425) | -0.35 (-1.25, 0.56) | < 0.001 | 0.46 |
| Adults | 13 (1263) | 0.04 (-0.26, 0.35) | < 0.001 | 0.78 |
| Caucasians | 12 (1086) | 0.15 (-0.02, 0.32) | 0.10 | 0.09 |
| T2DM patients | 3 (143) | 0.04 (-0.71, 0.79) | 0.02 | 0.91 |
| Overweight/obesity patients | 11 (986) | 0.02 (-0.33, 0.37) | < 0.001 | 0.91 |
| Insulin (CC + CG vs. GG) |  |  |  |  |
| All subjects | 16 (1879) | -0.01 (-0.21, 0.20) | < 0.001 | 0.95 |
| Females | 5 (454) | -0.08 (-0.47, 0.31) | 0.05 | 0.68 |
| Males | 3 (363) | 0.80 (-0.07, 1.67) | < 0.01 | 0.07 |
| Adults | 14 (1823) | -0.04 (-0.23, 0.15) | < 0.001 | 0.69 |
| Caucasians | 11 (1046) | 0.05 (-0.15, 0.26) | 0.02 | 0.61 |
| Asians | 5 (833) | 0.14 (-0.59, 0.32) | < 0.001 | 0.55 |
| Overweight/obesity patients | 9 (870) | -0.02 (-0.25, 0.21) | 0.02 | 0.86 |
| T2DM patients | 4 (443) | 0.09 (-0.26, 0.45) | 0.10 | 0.61 |
| General/control subjects | 4 (625) | 0.04 (-0.62, 0.70) | < 0.001 | 0.91 |
| HOMA-IR (CC + CG vs. GG) |  |  |  |  |
| All subjects | 9 (870) | -0.01 (-0.29, 0.28) | 0.001 | 0.96 |
| Caucasians | 8 (693) | 0.08 (-0.20, 0.35) | 0.01 | 0.59 |

*LEPR*, leptin receptor gene; SMD, standardized mean difference; 95% CI, 95% confidence interval; HOMA-IR, homeostasis model assessment of insulin resistance; T2DM, type 2 diabetes mellitus.

**Table S17.** Meta-analyses between *LEPR* rs1805094 variant and lipid-metabolism markers.

| Groups or subgroups | Comparisons (Subjects) | SMD (95% CI) | *P*_Heterogeneity_ | *P*_SMD_ |
| --- | --- | --- | --- | --- |
| Triglycerides (CC + CG vs. GG) |  |  |  |  |
| All subjects | 16 (1425) | -0.12 (-0.24, 0.01) | 0.39 | 0.07 |
| Females | 3 (370) | -0.20 (-0.56, 0.16） | 0.20 | 0.28 |
| Males | 3 (363) | 0.05 (-0.19, 0.29） | 0.43 | 0.67 |
| Adults | 12 (1241) | -0.10 (-0.22, 0.03) | 0.43 | 0.13 |
| Children/adolescents | 4 (184) | -0.22 (-0.71, 0.27） | 0.24 | 0.38 |
| Caucasians | 11 (1064) | -0.06 (-0.19, 0.07) | 0.65 | 0.37 |
| Asians | 3 (233) | -0.13 (-0.83, 0.58) | 0.16 | 0.72 |
| Overweight/obesity patients | 11 (986) | -0.17 (-0.31, -0.03) | 0.78 | 0.02 |
| General/control subjects | 4 (377) | -0.08 (-0.56, 0.39) | 0.13 | 0.73 |
| TC (CC + CG vs. GG) |  |  |  |  |
| All subjects | 14 (1297) | 0.05 (-0.09, 0.18) | 0.33 | 0.49 |
| Females | 3 (370) | -0.03 (-0.42, 0.35） | 0.16 | 0.87 |
| Males | 3 (363) | -0.07 (-0.31, 0.17） | 0.39 | 0.56 |
| Adults | 12 (1241) | 0.05 (-0.10, 0.20) | 0.20 | 0.50 |
| Asians | 3 (233) | -0.24 (-0.61, 0.13） | 0.76 | 0.21 |
| Caucasians | 11 (1064) | 0.08 (-0.06, 0.23) | 0.31 | 0.25 |
| Overweight/obesity patients | 10 (910) | 0.02 (-0.14, 0.18) | 0.31 | 0.81 |
| General/control subjects | 3 (325) | 0.002 (-0.26, 0.26) | 0.95 | 0.99 |
| LDL-C (CC + CG vs. GG) |  |  |  |  |
| All subjects | 15 (1363) | 0.02 (-0.12, 0.15) | 0.31 | 0.79 |
| Females | 3 (370) | -0.17 (-0.68, 0.34） | 0.05 | 0.51 |
| Males | 3 (363) | -0.02 (-0.26, 0.22） | 0.79 | 0.86 |
| Adults | 11 (1179) | 0.04 (-0.12, 0.21) | 0.13 | 0.61 |
| Children/adolescents | 4 (184) | -0.13 (-0.52, 0.26） | 0.93 | 0.52 |
| Caucasians | 10 (1002) | 0.09 (-0.05, 0.22) | 0.51 | 0.20 |
| Asians | 3 (233) | -0.43 (-0.81, -0.06) | 0.77 | 0.03 |
| Overweight/obesity patients | 11 (986) | 0.05 (-0.13, 0.23) | 0.13 | 0.61 |
| General/control subjects | 4 (377) | -0.03 (-0.27, 0.20) | 0.84 | 0.78 |
| HDL-C (CC + CG vs. GG) |  |  |  |  |
| All subjects | 15 (1363) | 0.001 (-0.18, 0.18) | 0.03 | 0.99 |
| Females | 3 (370) | 0.17 (-0.22, 0.57) | 0.15 | 0.39 |
| Males | 3 (363) | -0.33 (-0.56, -0.09) | 0.63 | < 0.01 |
| Adults | 11 (1179) | -0.07 (-0.22, 0.08) | 0.24 | 0.35 |
| Children/adolescents | 4 (184) | 0.21 (-0.48, 0.90) | 0.05 | 0.55 |
| Caucasians | 10 (1002) | -0.14 (-0.28, -0.01) | 0.85 | 0.04 |
| Asians | 3 (233) | -0.07 (-0.93, 0.79) | 0.08 | 0.87 |
| Overweight/obesity patients | 11 (986) | 0.05 (-0.14, 0.23) | 0.10 | 0.63 |
| General/control subjects | 4 (377) | -0.17 (-0.67, 0.33) | 0.10 | 0.50 |

*LEP*, leptin gene; SMD, standardized mean difference; 95% CI, 95% confidence interval; TC, total cholesterol; LDL-C, low-density lipoprotein cholesterol; HDL-C, high-density lipoprotein cholesterol.
